# Supplementary material for: Blood–brain barrier genetic disruption leads to protective barrier formation at the Glia Limitans
Source: PLoS Biol. 2020 Nov 30;18(11):e3000946. doi: 10.1371/journal.pbio.3000946 (PMC7728400; doi:10.1371/journal.pbio.3000946)
Supplement: S1 Text — Supporting information file containing the S1 Data and S2 Data legends and DOI links (A), the Supporting Methods (B), and the associated References (C). (DOCX) [file pbio.3000946.s001.docx]

**Supporting information**

**A. Supporting Datasets**

S1 Data: Prism spreadsheet containing, in separate sheets, the underlying numerical data and statistical analysis for Fig 1, C-J, Fig 2, A-C, Fig 2, H-L, Fig 3, C-F, Fig 3, I-K, Fig 4, C, G, H, Fig 5, A-D, Fig 5, I,J,N,O, Fig 6, A-D, Fig 6, F, Fig 7, A-D and Fig 7, F,H,J.

[https://doi.org/10.6084/m9.figshare.12625034.v6](https://doi.org/10.6084/m9.figshare.12625034.v4)

S2 Data: Excel and Prism spreadsheet containing, in separate sheets, the underlying numerical data and statistical analysis for S1 Fig, F, S2 Fig, B-C, S4 Fig, D, S6 Fig, C-D, and S7 Fig, A.

[https://doi.org/10.6084/m9.figshare.12625085.v7](https://doi.org/10.6084/m9.figshare.12625085.v6)

**B. Supporting Methods**

**Cadherin5-Cre^ERT2^;Rosa^mTmG^ mice**

*Rosa26^mTmG^* mice[1] and *Cadherin5-Cre^ERT2^* mice[2] were obtained from Jackson Laboratories. The Cre recombinase in *Cadherin5-Cre^ERT2^* mice was activated by intra-peritoneal injection of 1 mg tamoxifen for 5 consecutive days at 8 weeks of age. Successful and specific activation of the Cre recombinase was verified in the CNS of *Cadherin5-Cre^ERT2^; Rosa26^mTmG^* mice (Fig S3, A) at different time points (2 weeks, 4 weeks, 6 months and 1 year post tamoxifen injections).

**Propidium Iodide viability test**

CNS MECs from Dhh^ECKO^ and control mice were isolated as described in the section Method- Primary culture of mouse CNS micro vascular endothelial cells (CNS MECs). 7 days post seeding, the cells were washed 3 times (5 min) with PBS and incubated 5 min on ice with Propidium Iodide (2µg/mL) (Sigma Aldrich, St. Louis, MO, USA) and Hoechst 33342 (1/10000) (Invitrogen, Carlsbad, CA, USA) to label cell nuclei. Samples were examined using a Zeiss Microsystems confocal microscope ([Oberkochen, Germany)](https://www.google.com/search?client=firefox-b-e&sxsrf=ACYBGNSGG69OQ0ioQ4pM7AAjaqD819iCtw:1579180369591&q=Oberkochen&stick=H4sIAAAAAAAAAOPgE-LUz9U3MEwutChWAjNNTTPMy7S0spOt9POL0hPzMqsSSzLz81A4VhmpiSmFpYlFJalFxYtYufyTUouy85MzUvN2sDICAP8BmH9UAAAA&sa=X&ved=2ahUKEwjLz9ynmYjnAhWozYUKHalcDjcQmxMoATARegQIDRAH), and stacks were collected with *z* of 1 μm.

**In vitro permeability assay**

150000 cells were seeded in Transwell® inserts. The day after, PBS, VEGFA (10 ng/mL) (CliniSciences, Nanterre, France) or D-Mannitol (20 ng/µL) (Sigma Aldrich, Saint Louis, MI, USA) was added to the upper chamber for 6 hours. 0.5 mg/mL 70 kDa FITC-Dextran (Sigma Aldrich, St. Louis, MO, USA) was added to the upper chamber 5 hours and 30 min after treatment by the pro-permeability factors. FITC fluorescence in the lower chamber was measured 30 minutes later.

**In Situ Hybridization (ISH)**

Sense and antisense riboprobe for in situ hybridization were prepared from pGEM-T (Invitrogen, Carlsbad, CA, USA) plasmid containing a 514-bp or a 519-bp RT-PCR amplified fragment corresponding respectively to the open reading frame or to the 3'-untranslated region of *mDhh*. cRNA probes were labeled using digoxigenin-11-UTP using Genius Labeling and in situ hybridization was carried out on serial 5 µm sections of C57BL/6 adult mouse brain fixed in 4% paraformaldehyde using Detection Kit (Boehringer Mannheim, Germany) as previously described[3]. Serial 5-µm sections were fixed in 4% paraformaldehyde for 30 minutes, rehydrated, treated with proteinase K (20 µg/mL) for 15 minutes, and then acetylated with triethanolamine, dehydrated, and air dried. Hybridization was carried out at 50°C for 16 hours with 200 ng/mL of probe in a hybridization buffer containing 50% deionized formamide, 1× Denhardt’s solution, 4× SSC, 10% Dextran sulfate, and 0.4 mg/mL ssDNA. Sections were washed twice for 15 minutes in 2× SSC at room temperature, treated with RNase A (100 µg/mL) in 2× SSC at 37°C for 30 minutes, and then washed twice for 20 minutes in 2× SSC at 42°C and then at room temperature. The alkaline phosphatase–conjugated anti-digoxigenin antibody (1:500) was incubated for 2 hours in 10% horse serum at room temperature, and the sections were rinsed overnight. The sections were incubated for 8 hours with the substrate nitroblue tetrazolium salt X-phosphate.

**In vivo permeability assay**

To quantify vascular permeability, mice were injected in the tail vein with 50 μL (25 mg/mL) 70 kDa FITC dextran (Sigma Aldrich, St. Louis, MO, USA) 30 minutes before sacrifice. The head was then decapitated, and the brain was removed and placed in a tube containing methanol for 24 hours; the brain was then embedded in paraffin. Sections of 7 μm were then cut for visualization.

**Microcomputed tomography analysis**

The brain vasculature was imaged with a high-resolution micro-CT imaging system (Bruker MicroCT, Kontich, Belgium), set to an effective detector pixel size of 0.08 mm. The apparatus used for imaging the vessels was the Bruker SkyScan microCT scanner from Bruker with spatial resolutions of 7 to 36 μm and used with the Bruker Acquisition and DigiXCT reconstruction utility programs from Digisens. Data were acquired in axial mode, as described previously(1). The volume was visualized with the Avizo program (ThermoFisher Scientific, Waltham, MS, USA).

**Flow cytometry**

C57BL/6 mice induced with EAE MOG_35-55_ *v*s placebo were sacrificed at day 13 post EAE induction. Spinal cords were collected and neurovascular units were isolated following the protocol described in the section Method-Isolation of neurovascular units from mouse CNS. The isolated neurovascular unit pellet was washed 3 times with the buffer B ((HBSS 1X Ca^2+^/ Mg^2+^ free with phenol red (Gibco, Waltham, MA, USA) and transferred in an enzyme solution (2 mg/mL Collagenase/Dispase (Roche, Bale, Switzerland), 0,147 µg/mL TLCK (Lonza, Bäle, Switzerland) and 10 µg/mL DNAse 1 (Roche, Bäle, Switzerland)), pre-warmed at 37 °C, before being placed on a shaking table at maximum speed agitation at 37 °C. After 30 minutes, the digestion was stopped by adding 10 mL of buffer B, the cell suspension centrifuged and the digested neurovascular pellet washed 3 times with 3 mL of buffer B. After the 3 washing steps, the digested neurovascular suspension was depleted in mouse CD45^+^ leukocytes using a MACS mouse CD45+ Cell Isolation Kit (Miltenyi Biotec, Paris, France) according to the manufacturer’s instructions. Cell suspension was then collected, incubated in blood cell lysis buffer (RBC lysis buffer) (BioLegend, San Diego, CA, USA) for 2 minutes at room temperature, and washed with PBS. Cells from spinal cords were incubated with Fc Blocker (BioLegend, San Diego, CA, USA) for 15 minutes at RT. Surface staining was performed in the dark for 30 minutes at 4°C with the APC-conjugated anti-PECAM1 antibody (eBioscience, San Diego, CA, USA) and/or PE/Cy7-conjugated anti-CD45 antibody (BioLegend, San Diego, CA, USA) in cell staining buffer (BioLegend, San Diego, CA, USA). Cells were washed twice and resuspended in cell staining buffer and DAPI. Single staining of tissue samples was used. Forward scatter and side scatter were used to gate cells excluding debris and cell aggregates, and PECAM1^+^ and CD45^+^ cells were gated on the DAPI^+^ live cell population. Samples were analyzed on an Accuri C6 flow cytometer (BD Biosciences, Franklin Lakes, NJ, USA). Data were interpreted using BD Accuri C6 Analysis Software.

**C. References**

1. Muzumdar MD, Tasic B, Miyamichi K, Li L, Luo L. A global double-fluorescent Cre reporter mouse. genesis. 2007;45(9):593‑605.

2. Azzoni E, Conti V, Campana L, Dellavalle A, Adams RH, Cossu G, Brunelli S. Hemogenic endothelium generates mesoangioblasts that contribute to several mesodermal lineages in vivo. Development. 1 mai 2014;141(9):1821‑34.

3. Duplàa C, Jaspard B, Moreau C, D’Amore PA. Identification and Cloning of a Secreted Protein Related to the Cysteine-Rich Domain of Frizzled: Evidence for a Role in Endothelial Cell Growth Control. Circ Res. 25 juin 1999;84(12):1433‑45.
