# Supplementary material for: Blood–brain barrier genetic disruption leads to protective barrier formation at the Glia Limitans
Source: PLoS Biol. 2020 Nov 30;18(11):e3000946. doi: 10.1371/journal.pbio.3000946 (PMC7728400; doi:10.1371/journal.pbio.3000946)

Fig 2 (panel G)

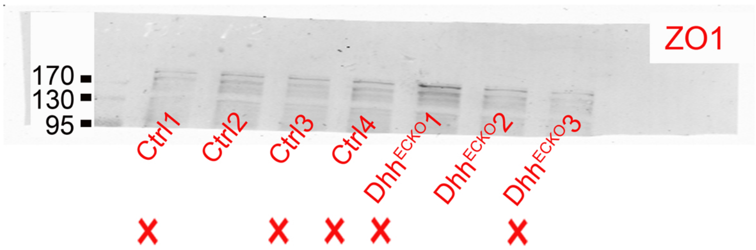

Fig 2 (panel G)

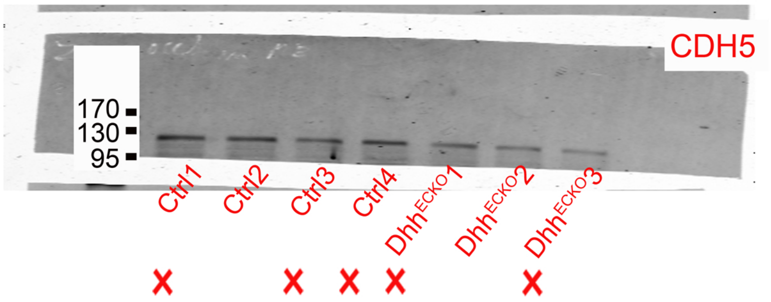

Fig 2 (panel G)

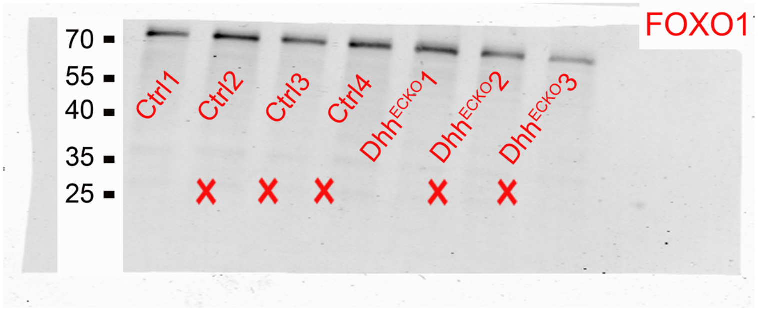

Fig 2 (panel G)

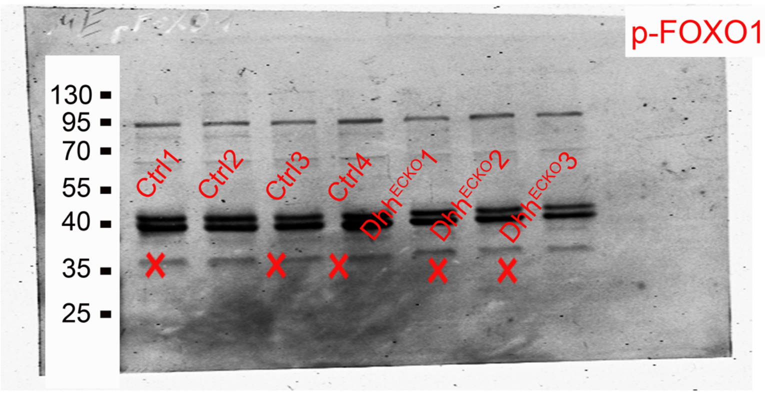

Fig 2 (panel G)

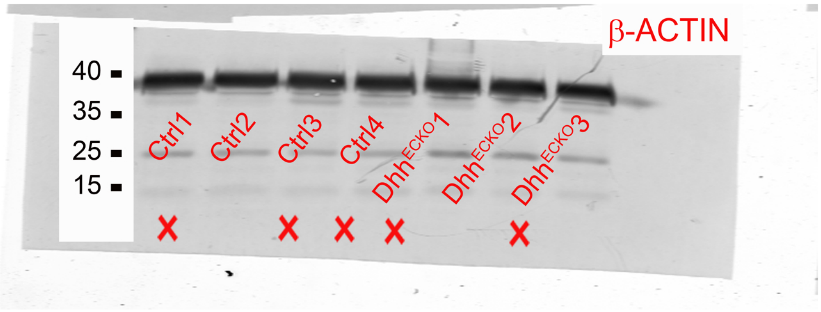

Fig 4 (panel F)

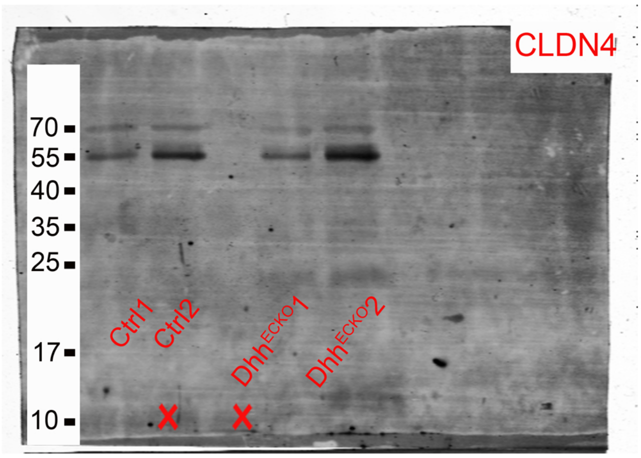

Fig 4 (panel F)

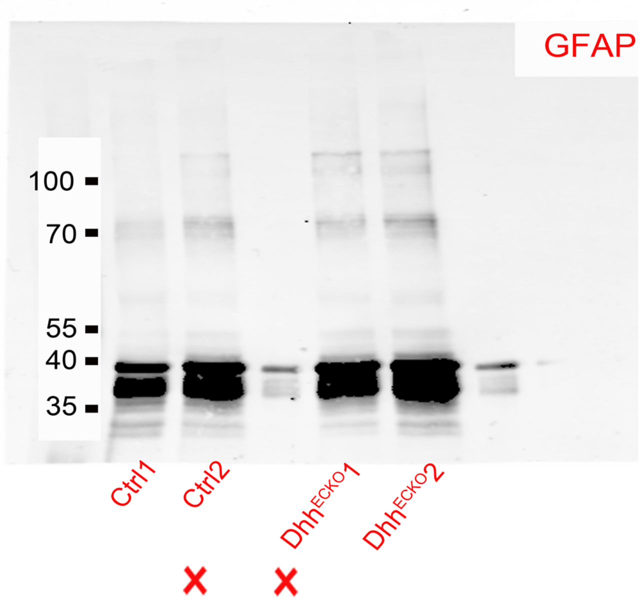

Fig 4 (panel F)

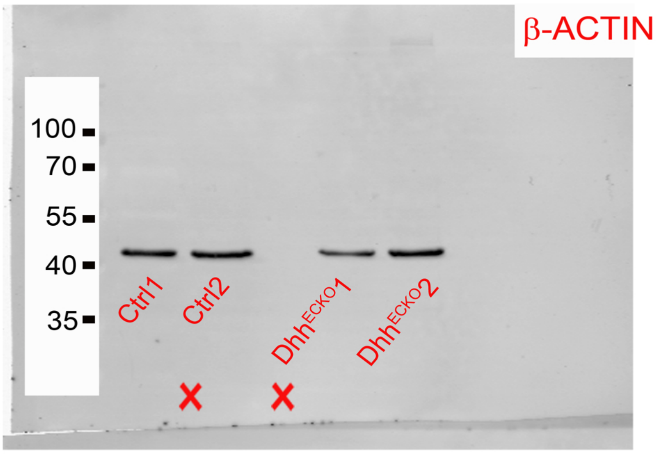

Fig 7 (panel I)

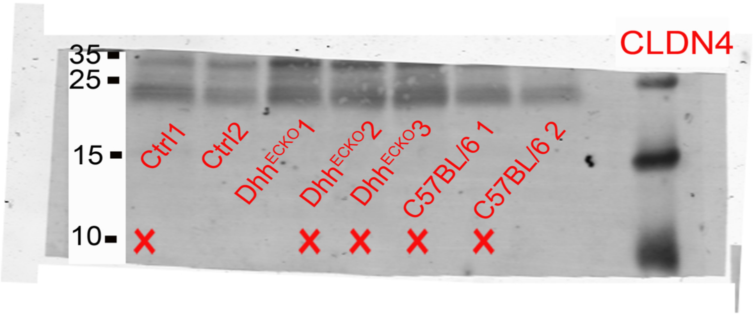

Fig 7 (panel I)

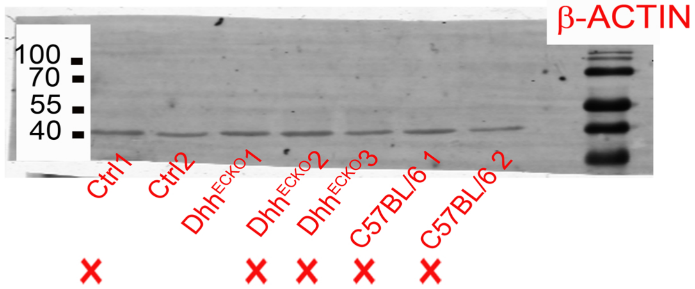

S6 Fig (panel A)

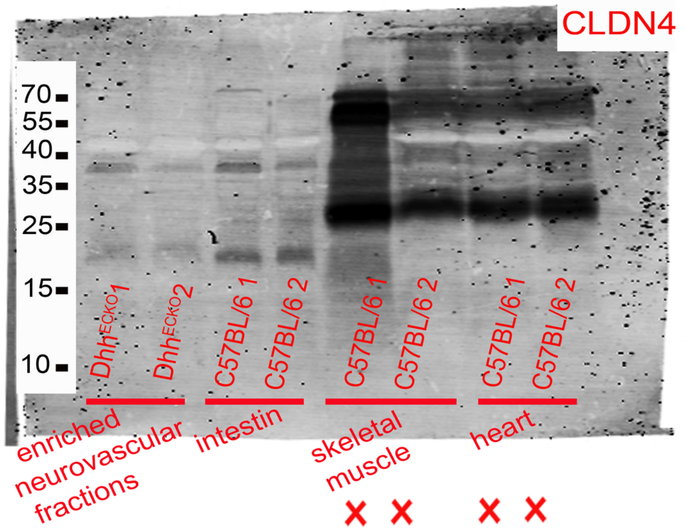

S6 Fig (panel A)

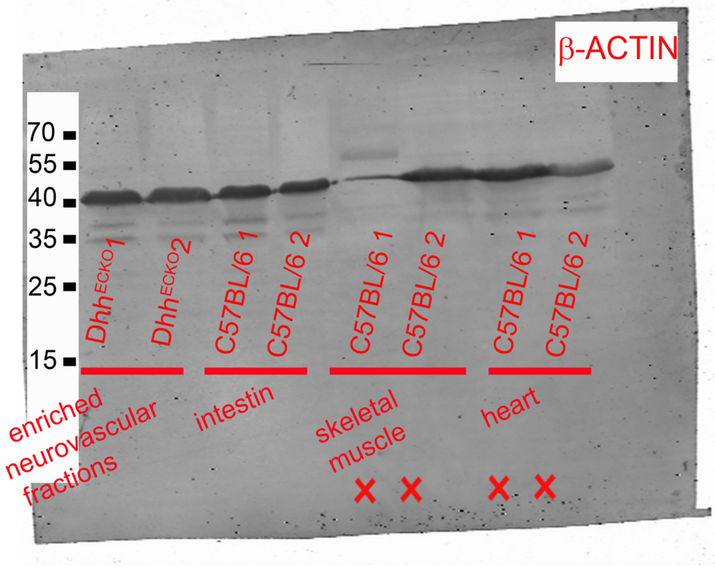

S6 Fig (panel B)

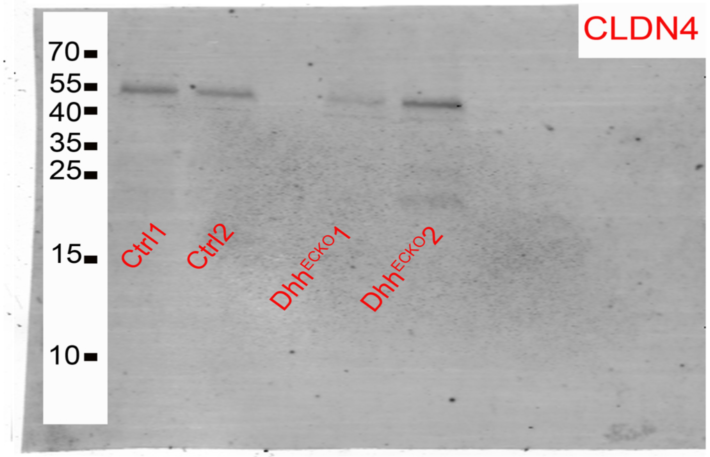

S6 Fig (panel B)

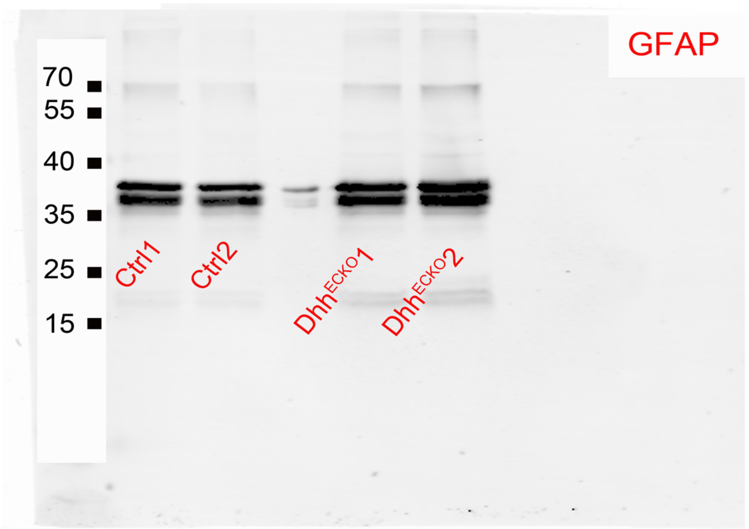

S6 Fig (panel B)

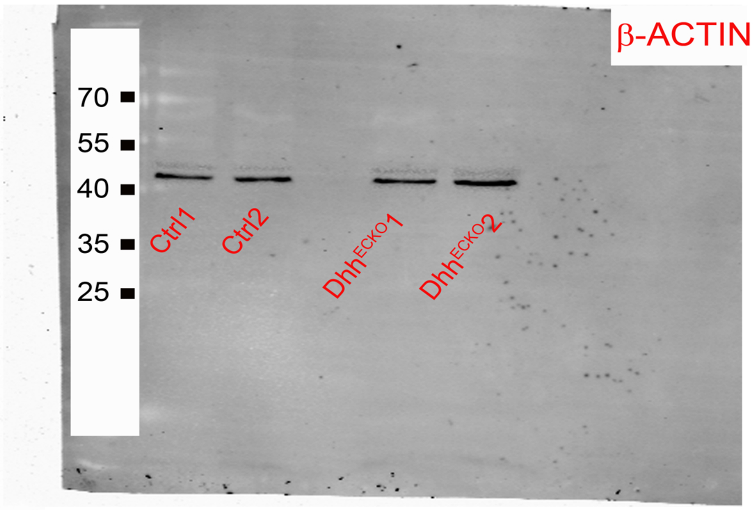

Supplement: S1 Raw Images — Supporting information file containing the original, uncropped, and minimally adjusted images supporting all blot and gel results reported in Fig 2 panel G, Fig 4 panel F, and Fig 7 panel I as well as S6 Fig panel A and B. (PDF) [file pbio.3000946.s002.pdf]
