# Supplementary figures and images for: Blood–brain barrier genetic disruption leads to protective barrier formation at the Glia Limitans
Source: PLoS Biol. 2020 Nov 30;18(11):e3000946. doi: 10.1371/journal.pbio.3000946 (PMC7728400; doi:10.1371/journal.pbio.3000946)

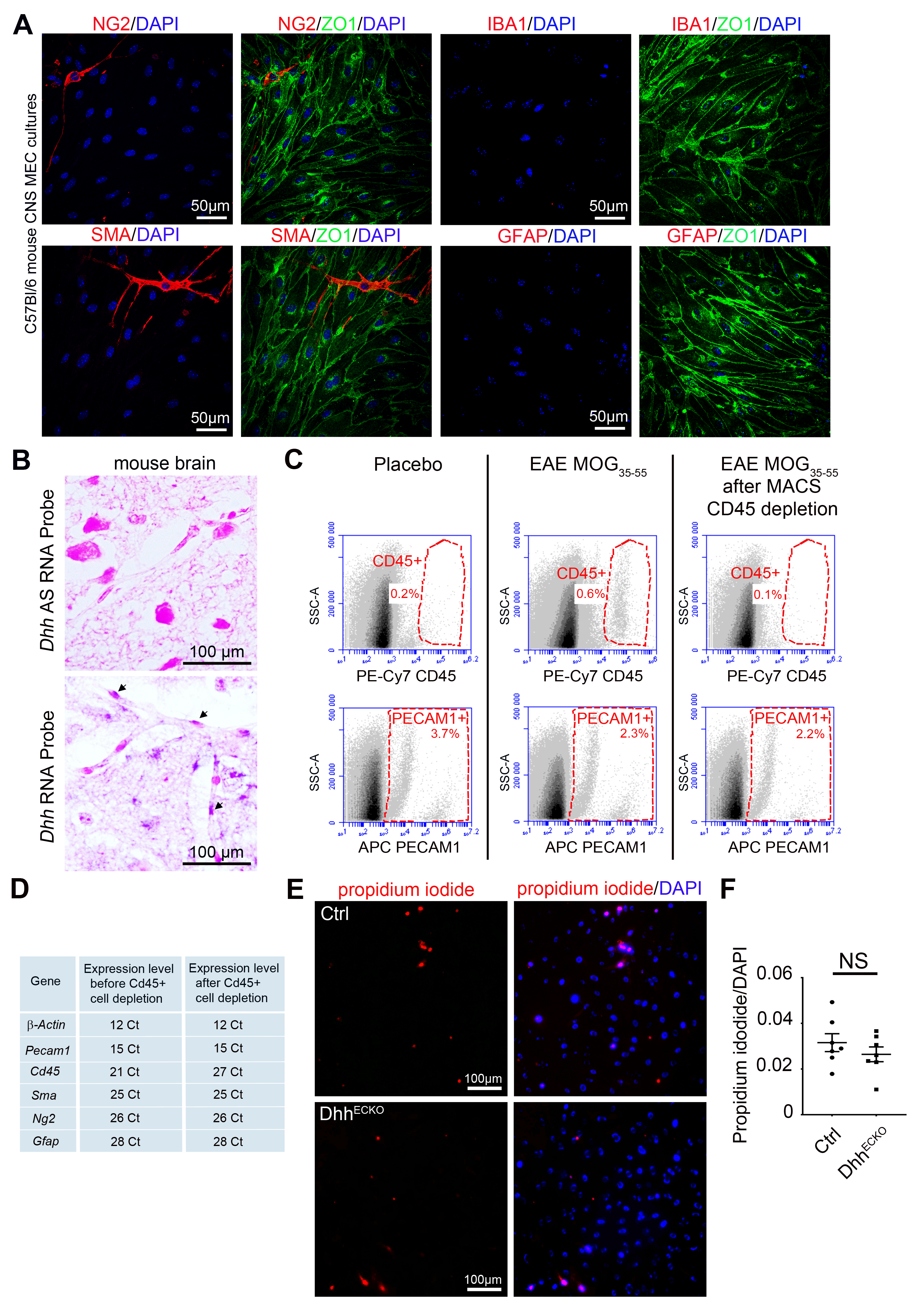

Supplement: S1 Fig — BMEC purity analysis shows limited contamination by other neurovascular components (A) Primary BMECs from DhhECKO and control mice were isolated and cultured on Lab-Tek. ZO1 (in green) and SMA, NG2, IBA1, or GFAP (in red) localizations were evaluated by immunofluorescent staining of a confluent cell monolayer. Nuclei were stained with DAPI (in blue). The experiment was repeated 3×. Mouse brain in situ hybridization analysis highlights Dhh expression at the BBB. (B) C57BL/6 cortical cross section hybridized with the Dhh RNA probe show Dhh expression in blood vessels. A control section hybridized with the Dhh antisense RNA probe show the absence of hybridization signals. MECs isolated from the spinal cord of mice induced with EAE MOG35-55 are highly contaminated by leukocytes (C) Spinal cord MECs were isolated from 12-week-old C57Bl/6 mice at day 13 post induction with EAE MOG35-55 or placebo and Pecam1, Cd45, Sma, Ng2, and Gfap expressions were quantified by qRT-PCR (cycle threshold mean values) before and after the cell suspension were depleted in CD45+ leukocytes. β-actin is used as a reference. A MACS CD45+ cell depletion step is sufficient to eliminate leukocyte contamination in MECs isolated from the spinal cord of mice induced with EAE MOG35-55 (D) Representative graphs of flow cytometry analysis performed on C57BL/6 mouse spinal cord MECs harvested at day 13 post EAE MOG35-55 induction. Analysis showed no CD45+ cell population in samples depleted in leukocytes using the MACS CD45+ cell isolation kit (Miltenyi Biotec) (EAE MOG35-55 spinal cord MECs n = 3; EAE MOG35-55 spinal cord MECs + MACS CD45+ cell isolation n = 3). Dhh endothelial knockdown does not impact CNS MEC viability in culture (E-F). (E) Primary CNS MECs from DhhECKO and control mice were isolated and cultured on Lab-Tek and immunostained with Propidium Iodide (in red) and Hoechst 33342 to label the nuclei (in blue). (F) DhhECKO versus control primary CNS MEC viability was evaluated by quantifying [file pbio.3000946.s003.tif]

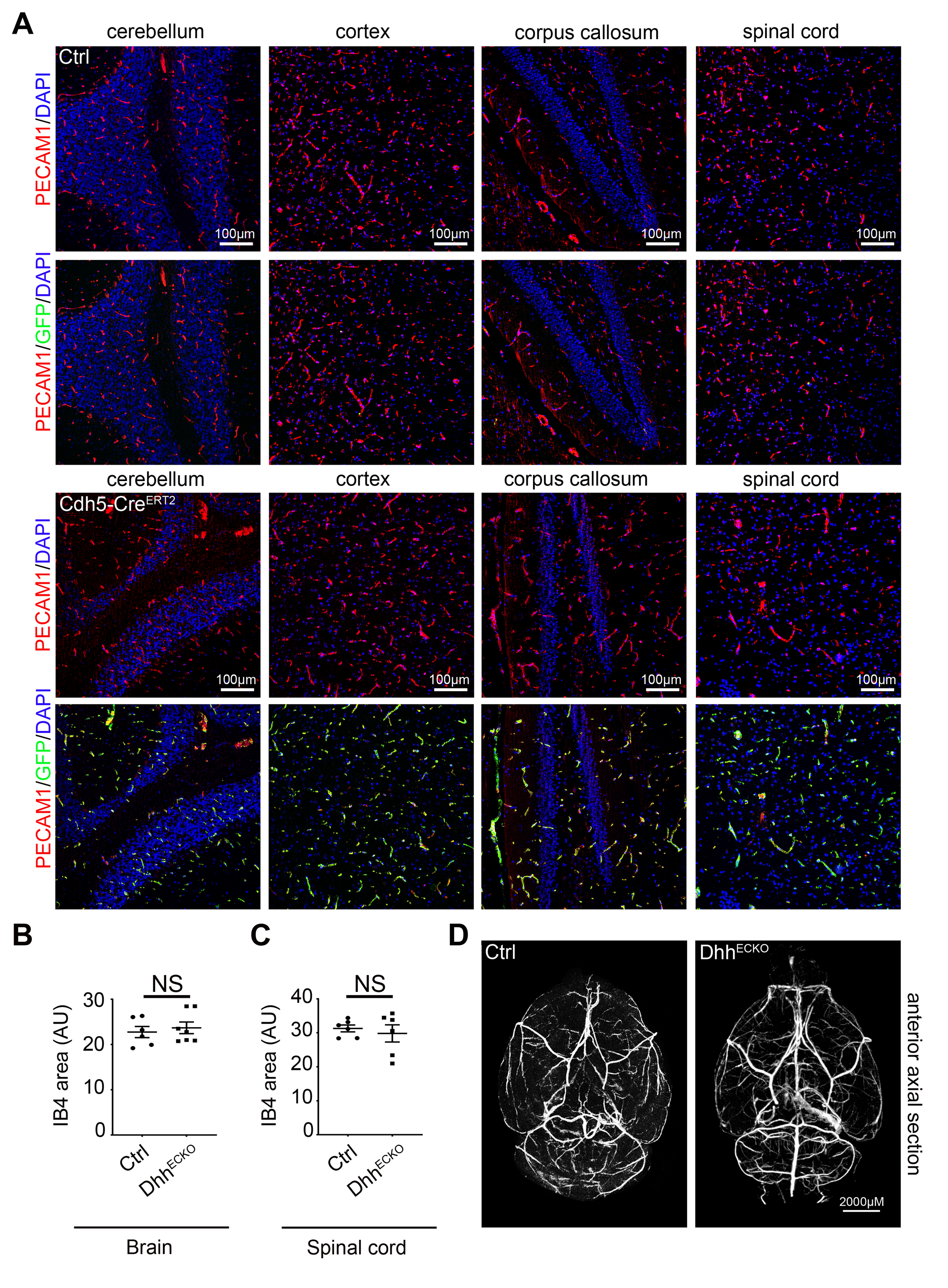

Supplement: S2 Fig — Cadherin5CreERT2 recombinase activation in blood vessels is successful and specific (A) Brain and spinal cord sections were harvested from Cadherin5CreERT2,Rosa26mTmG mice and littermate controls and immunostained with anti-GFP (in green) and anti-PECAM1 (in red) antibodies. Dhh endothelial knockdown does not impact CNS angiogenesis (B) Spinal cord sections were harvested from DhhECKO mice and littermate controls and immunostained with an anti-IB4 (in green) antibody. IB4 positive area was quantified (DhhECKO n = 7, control n = 6). (C) Cortical sections were harvested from DhhECKO mice and littermate controls and immunostained with an anti-IB4 (in green) antibody. IB4 positive area was quantified (DhhECKO n = 6, WT n = 6). Dhh endothelial knockdown does not impact brain angioarchitecture (D) The vascular network in the brain of DhhECKO mice and control littermates was imaged by microcomputed tomography (micro-CT). NS, Mann–Whitney U test. The underlying data for S2 Fig can be found in S1 Data (individual numerical data (excel file)) and S2 Data (statistical analysis (Prism file)) (https://doi.org/10.6084/m9.figshare.12625034.v6; https://doi.org/10.6084/m9.figshare.12625085.v7). (TIF) [file pbio.3000946.s004.tif]

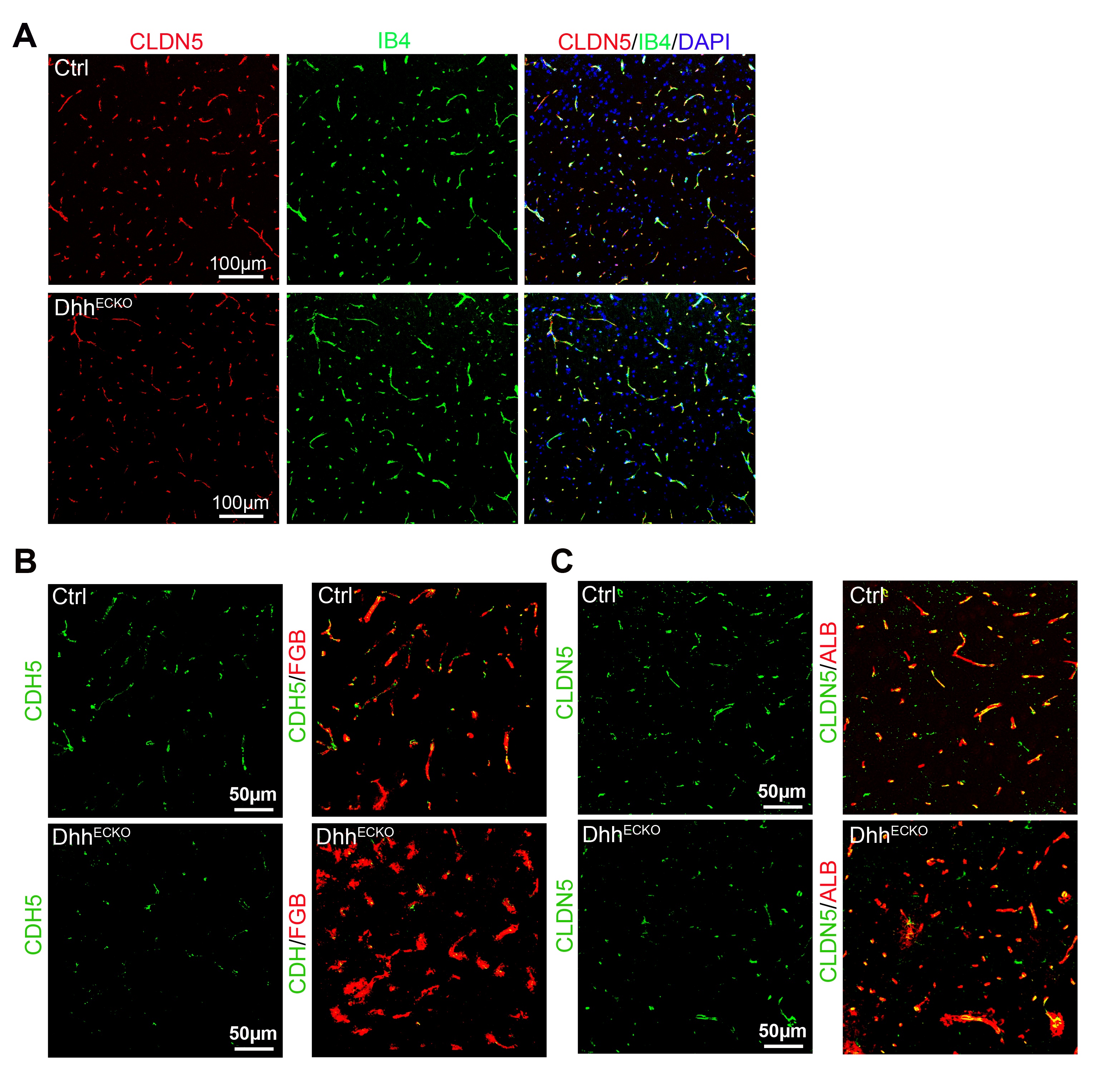

Supplement: S3 Fig — (A) Spinal cord sections were harvested from DhhECKO mice and littermate controls and immunostained with anti-IB4 (in green) and anti-CLDN5 (in red) antibodies. Representative IB4/CLDN5 staining was shown. (B–C) Spinal cord sections were harvested from DhhECKO mice and littermate controls and immunostained with anti-CDH5 or anti-CLDN5 (in green), and anti-FGB or anti-ALB (in red) antibodies. Representative (B) CDH5/FGB and (C) CLDN5/ALB staining were shown. (TIF) [file pbio.3000946.s005.tif]

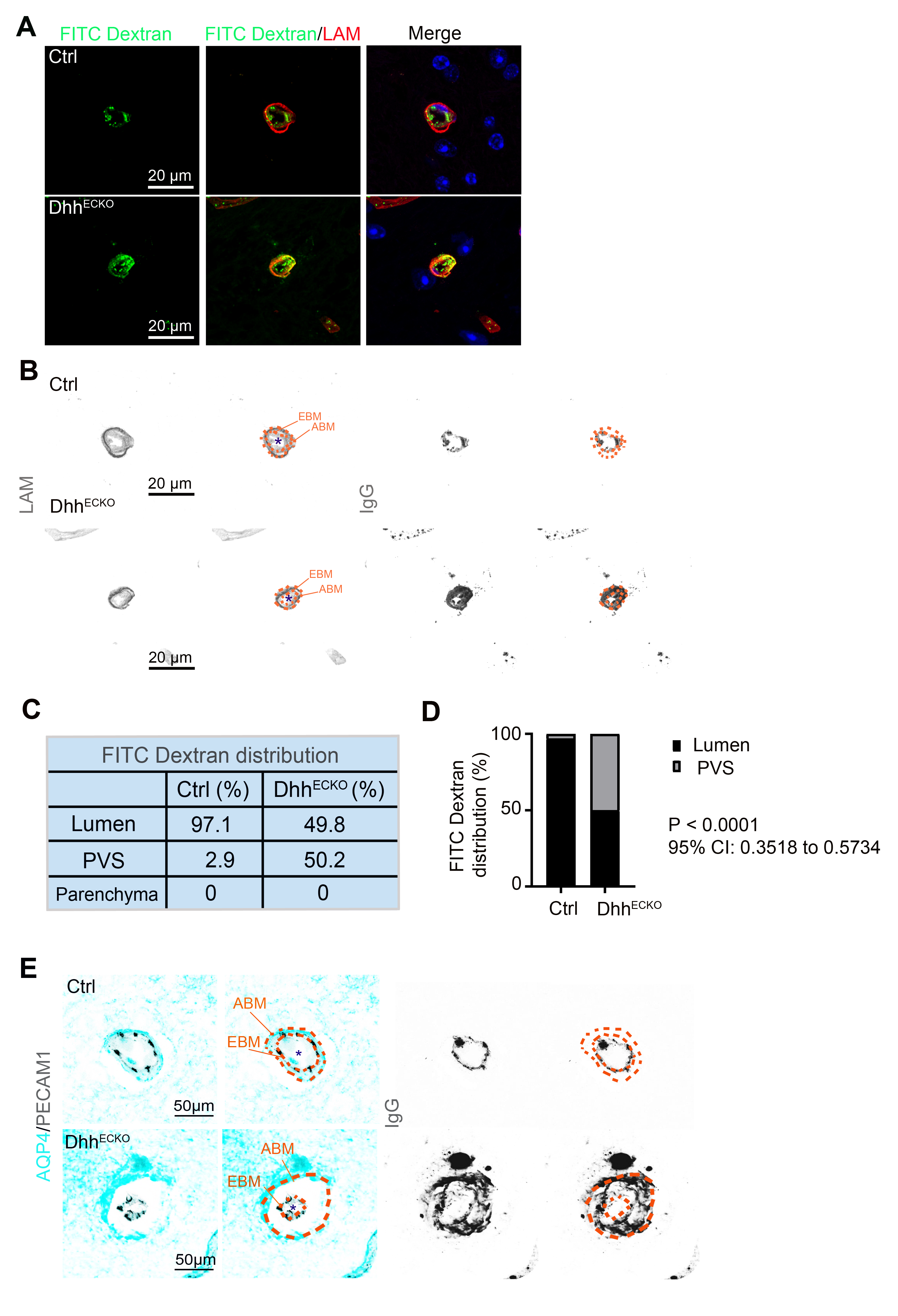

Supplement: S4 Fig — DhhECKO-induced BBB breakdown is sufficient to induce a secondary CNS protective barrier at the Glia Limitans. (A) Brain sections were harvested from DhhECKO mice and littermate controls injected with 70 kDa FITC Dextran and (A) immunostained with an anti-LAM (in red) antibody (nuclei were stained with DAPI (in blue)). Representative LAM/FITC Dextran staining was shown. (B) Negative working images of LAM channels were used to highlight the endothelial (EBM) and astrocyte (ABM) basement membranes, using orange dotted lines. The outlines were then transferred to the FITC Dextran images to discriminate the distribution of FITC Dextran between the lumen, PVS, and parenchyma. (C–D) The distribution of FITC Dextran within the lumen, PVS, and parenchyma was quantified. (DhhECKO n = 6, control n = 6) P < 0.0001, 95% CI: 0.3518 to 0.5734, chi-squared test. (E) Negative working images of AQP4/PECAM1 channels were used to highlight the endothelial (EBM) and astrocyte (ABM) basement membranes, using orange dotted lines. The outlines were then transferred to the IgG images to discriminate the distribution of IgG between the lumen, PVS, and parenchyma. The underlying data for S4 Fig can be found in S2 Data (https://doi.org/10.6084/m9.figshare.12625085.v7). (TIF) [file pbio.3000946.s006.tif]

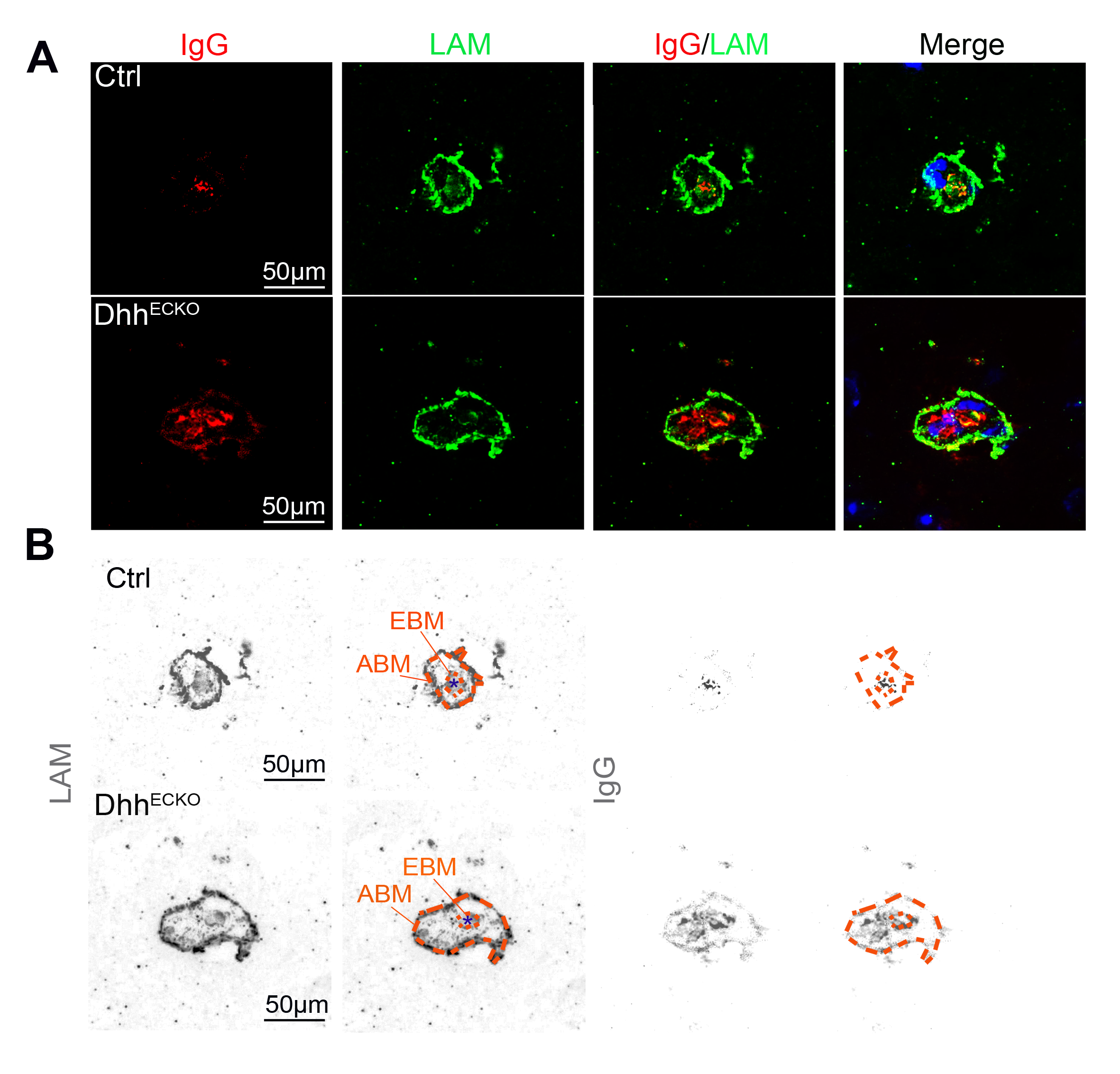

Supplement: S5 Fig — DhhECKO-induced BBB breakdown is sufficient to induce a secondary CNS protective barrier at the Glia Limitans. (A) Spinal cord sections were harvested from DhhECKO mice and littermate controls and immunostained with anti-LAM (in green) and anti-IgG (in red) antibodies (nuclei were stained with DAPI (in blue)). Representative LAM/IgG staining was shown. (B) Negative working images of LAM channels were used to highlight the endothelial (EBM) and astrocyte (ABM) basement membranes, using orange dotted lines. The outlines were then transferred to the IgG images to discriminate the distribution of IgG between the lumen, PVS, and parenchyma. (TIF) [file pbio.3000946.s007.tif]

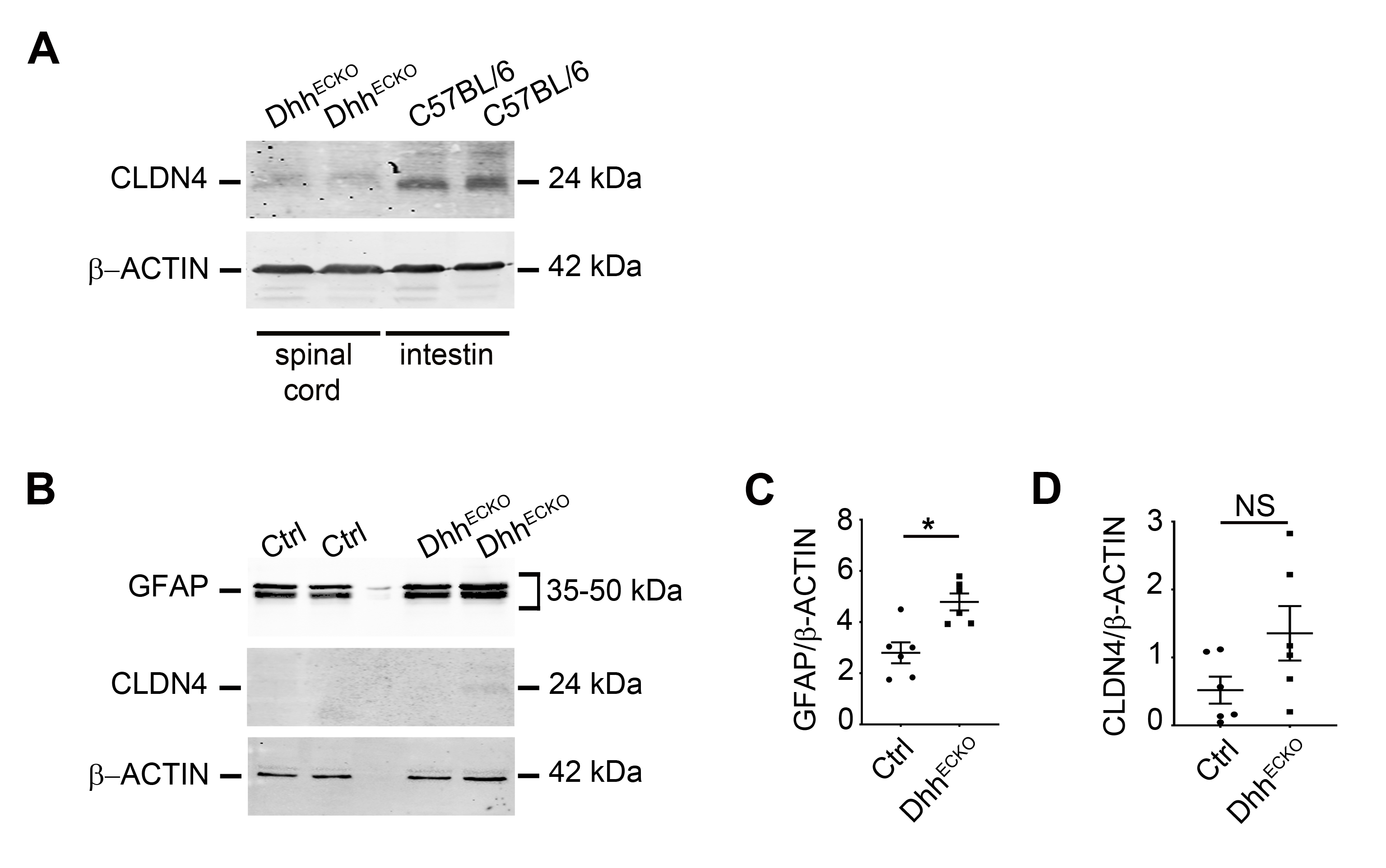

Supplement: S6 Fig — Small intestine samples are used as a positive control for the quantification of CLDN4 expression by western blot. (A) Representative blots of CLDN4 expression level on control mouse neurovascular unit lysates and mouse small intestine lysates were shown. There is astrocyte reactivity but no astrocytic CLDN4 up-regulation at the capillary level in DhhECKO mouse CNS. (B) Representative blots of GFAP and CLDN4 expression level on DhhECKO and control lysates were shown. Lysates were obtained with neurovascular units, which are 20 μm and larger. (C) GFAP expression level was quantified by western blot on DhhECKO and control lysates obtained with neurovascular units, which are 20 μm and larger. (D) CLDN4 expression level was quantified by western blot on DhhECKO and control lysates obtained with neurovascular units, which are 20 μm and larger. (DhhECKO n = 6, WT n = 6). *P ≤ 0.05, Mann–Whitney U test. The underlying data for S6 Fig can be found in S2 Data (https://doi.org/10.6084/m9.figshare.12625085.v7). (TIF) [file pbio.3000946.s008.tif]

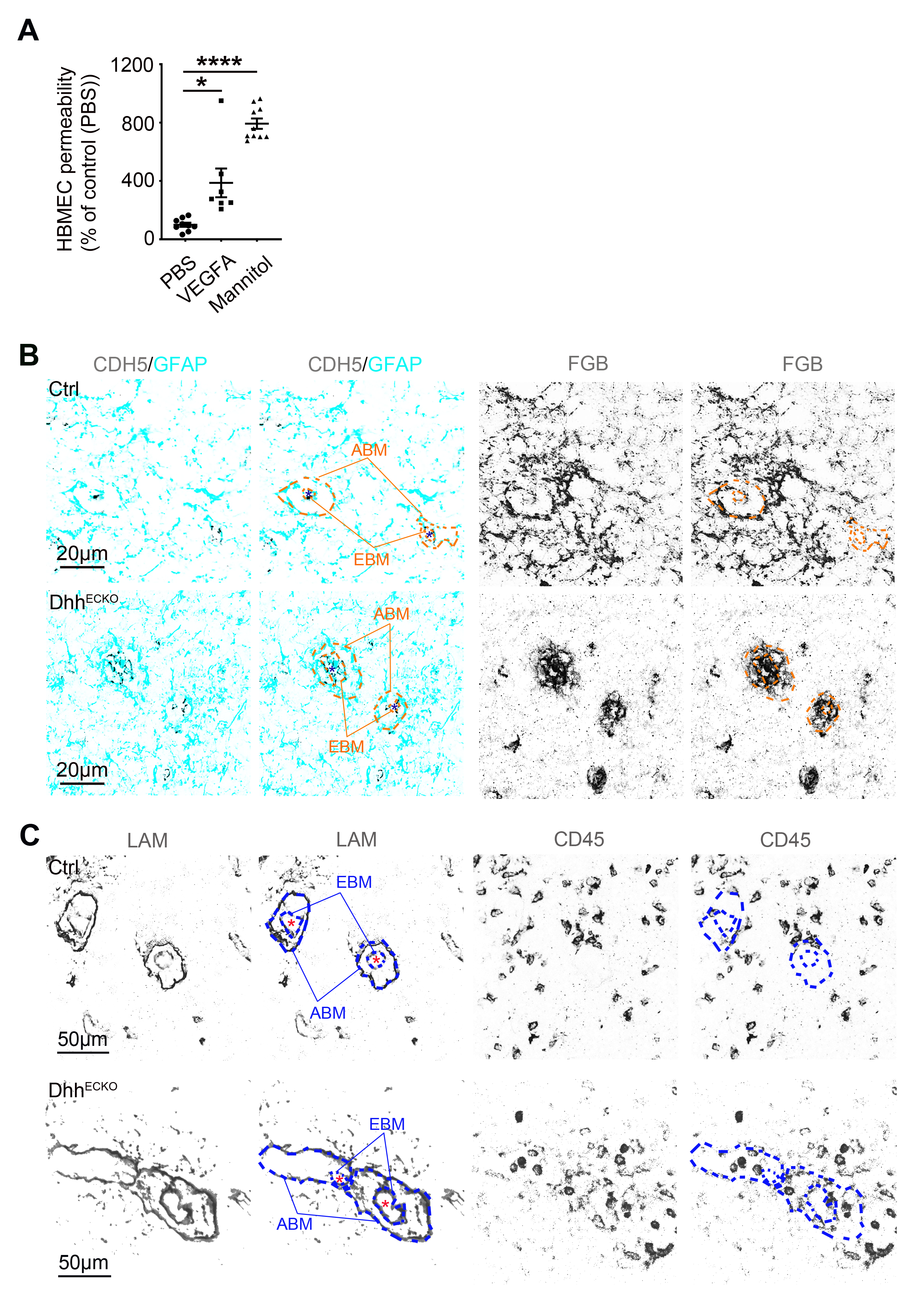

Supplement: S7 Fig — Both VEGFA and Mannitol induce HBMEC permeability in vitro. (A) Cultured HBMECs were treated with PBS, VEGFA, or Mannitol for 6 h, and HBMEC monolayer permeability to 70 kDa FITC Dextran was quantified. Mice with endothelial Dhh knockdown display a reinforced barrier at the Glia Limitans restraining access to the parenchyma to inflammation in a model of multiple sclerosis: (B) Negative working images of GFAP/CDH5 channels were used to highlight the endothelial (EBM) and astrocyte (ABM) basement membranes, using orange dotted lines. The outlines were then transferred to the FGB images to discriminate the distribution of FGB between the lumen, PVS, and parenchyma. (C) Negative working images of the LAM channel were used to highlight the endothelial (EBM) and astrocyte (ABM) basement membranes, using blue dotted lines. The outlines were then transferred to the CD45 images to discriminate the distribution of leukocytes between the lumen, PVS, and parenchyma. *P ≤ 0.05, ****P ≤ 0.0001 Kruskal–Wallis test. The underlying data for S7 Fig can be found in S2 Data (https://doi.org/10.6084/m9.figshare.12625085.v7). (TIF) [file pbio.3000946.s009.tif]
