# Supplementary material for: Blood–brain barrier genetic disruption leads to protective barrier formation at the Glia Limitans
Source: PLoS Biol. 2020 Nov 30;18(11):e3000946. doi: 10.1371/journal.pbio.3000946 (PMC7728400; doi:10.1371/journal.pbio.3000946)
Supplement: Attachment — Submitted filename: response to reviewers.docx [file pbio.3000946.s010.docx]

Reviewer #1:

Previous work from other labs have highlighted the concept of the double blood brain barrier or more accurately, the double-basement membrane structure of cerebral blood vessels (endothelial basement membrane and parenchymal basement membrane made by the glia limitans). Results suggested that penetration of the endothelial and parenchymal barriers are independent steps involving distinct molecular mechanisms.

In this study, the authors investigated this concept in a mouse model with conditional inactivation of Desert Hedghog (Dhh) in basal condition and after immunization by MOG, as a model of experimental encephalomyelitis. Although the concept is interesting, there are several major issues which limit the significance of this paper.

Figure 1:

On panel A, positive and negative controls are missing to assess the purity of the preparation and confirm that these are endothelial cells.

CNS MEC culture purity was assessed (7 days post seeding) by quantifying *Pecam1* mRNA expression level as a positive vascular marker and *Sma*, *Ng2*, *Cd45* and *Gfap* mRNA expression level to assess smooth muscle cell, pericyte, leukocyte and astrocyte contaminations respectively (Fig 1, A). Additionally, we performed immunostaining on primary CNS MEC cultures using ZO1 to identify the endothelial cell layer and NG2, SMA, GFAP and IBA1 to measure pericyte, smooth muscle cell, astrocyte and microglia contaminations (Fig S1, A). As expected *Pecam1* and ZO1 expressions are predominant in the CNS MEC preparations; however there is a low contamination by other components of the neurovascular unit especially by pericytes and smooth muscle cells.

The manuscript has been edited to annex this information (Line 163 to Line 166).

The authors jump throughout the paper from murine to human cells without any justification. Please explain.

Primary mouse CNS MECs remain our leading choice in this paper.

However, to bring a translational aspect to the study, we used HBMECs to confirm that *DHH* expression level is down-regulated under inflammatory condition in human endothelial cells (Fig 1, C-E).

We also relied on HBMEC and NHA cultures to analyze astrocyte response to endothelial signals *in vitro* (Fig 5, A-J) because this experiment required a significant amount of cellular material which would have been difficult to obtain from C57Bl/6 mice.

Additionally, we are experiencing difficulties to isolate adult mouse astrocytes in the laboratory.

In situ hybridization on murine brain sections is needed to confirm production of Dhh by endothelial cells.

Dhh endothelial expression in mouse brain, using In Situ Hybridization, has been added to the paper (Fig S1, B).

The manuscript has been edited to annex this information (Line 166 to Line 167).

On panels F-H, it is unclear whether expression has been analyzed in isolated microvessels or in cultured brain endothelial cells. Authors analyzed expression of Dhh 16 days after MOG immunization, which is quite late. Please explain why this late time point has been selected.

To analyze the expression level of *Dhh*, *Icam1*, *Vcam1*, *Cldn5* and *Zo1* in C57Bl/6 mice induced with EAE MOG_35-55_ *vs* placebo (Fig 1, F-J), we isolated spinal cord micro-vessels (using a gradient of Dextran) which underwent a digestion step followed by a CD45^+^ cell depletion step using a MACS CD45+ cell isolation kit (Miltenyi Biotec) to discard the contamination by inflammatory cells induced by EAE (Fig S1, C-D).

The first time we did this analysis, we chose day 16 post EAE induction to measure *Dhh* expression level, which corresponds to the late phase of symptom progression in the animals, just before they reach the EAE plateau phase. The experiment has been redone, and this time, we did the analysis at day 13 post EAE induction, five days after the onset of the first clinical symptoms. In both cases we observed the down-regulation of *Dhh* expression in isolated spinal cord vessels (Fig 1, F).

The manuscript has been edited to annex this information (Line 174 to Line 177).

Figure 2 and 3

The conclusion that expression of VE-cadherin and claudin 5 is decreased in Dhh KO mice is unfortunately not convincing.

Figure 2: There is a discrepancy between immunostaining (panels D-F) and qRT-PCR or Western blot data, regarding the expression of VE-cadherin and claudin 5. IF pictures show an increased expression of VE-cadherin and claudin 5 in mutant cells whereas qRT-PCR and WB quantifications show the opposite.

Mouse CNS MEC culture immunostaining were only used as a qualitative tool to show tight junction disorganization (Fig 2, D-F). As previously described by A.G Arroyo’s lab in 2014(1), what we were looking at is the breadth of CDH5 and CLDN5 at junctions. In controls, CDH5 and CLDN5 displayed a well-defined pattern of sharp contours at endothelial cell-cell contacts. By contrast, in Dhh^ECKO^ cultures, a broader, more irregular pattern was detected at endothelial cell-cell contacts (Fig 2, D and F). Because we have a broadened distribution and wider peak of CDH5 and CLDN5 intensity in Dhh^ECKO^ cells compared to controls doesn’t mean that their expression level is increased. On the contrary, it is a reflection of the endothelial CNS barrier dysfunction. This is why we are using qRT-PCR and western blot analysis as quantitative tools to measure CDH5 and CLDN5 expression levels in Dhh^ECKO^ and control CNS MECs (Fig 2, B and G).

The manuscript has been modified to better explain what we are looking at on mouse CNS MEC labtek immunostaining (Line 213 to Line 216).

Moreover, what is the genotype of control mice? Were these mice treated with tamoxifen? Also, same comment as above regarding brain endothelial cells vs isolated microvessels.

In all Fig 2 panels, the control (Ctrl) group refers to CNS MECs isolated from Dhh^Flox/Flox^ mice which were injected with tamoxifen by intraperitoneal injection of 1 mg tamoxifen for 5 consecutive days at 8 weeks of age, just like the Dhh^ECKO^ group.

The paragraph “To do so, we used isolated CNS MECs from *Cdh5-Cre^ERT2^;Dhh^Flox/Flox^* mice (Dhh^ECKO^ mice) and *Dhh^Flox/Flox^* control littermates, two weeks after inducing the knockdown by intra-peritoneal injection of tamoxifen. There is no difference between Dhh^ECKO^ and control mouse cell culture viability” has been added to the manuscript (Line 208 to Line 209). The term “CNS MECs” refers to primary cultures of brain and spinal cord (CNS) microvascular endothelial cells (Line 211) (as described in the Methods: line 682 to line 697).

Figure 3: On panels A -C, what is missing is a vascular marker to quantify claudin5 and VE-cadherin area over the vessel area, to normalize for the number and length of vessels.

Isolectin B4 (IB4)-CDH5 (Fig 3, A) and IB4-CLDN5 (Fig S3, A) co-immunostaining have been performed and CDH5 and CLDN5 (Fig 3, C-D) areas over the vascular area have been quantified to normalize for the number and length of blood vessels.

The manuscript has been edited to annex this information (Line 270 to Line 272).

(Line 213 to Line 216)

Methodology to analyze the BBB integrity in Dhh KO mice is not appropriate. At least mice must be transcardially perfused to remove intravascular plasma proteins which can passively diffuse in the vessel wall even after fixation (see Mestre et al, Nature Communications 201).

To analyze BBB integrity in Dhh^ECKO^ mice *vs* control littermates, mouse brain and spinal cord tissues were harvested. These tissues originate from mice which have been transcardially perfused with PBS (10mL) followed by 10% Formalin (10mL) to remove intravascular plasma proteins.

We clarified this protocol in the Methods (Line 776 to Line 778).

Moreover, because quantification of endogenous fibrinogen or albumin on sections can be challenging and is subjective, analysis of exogenous tracers such as cadaverine of Dextran is requested.

The vascular extravasation of 70 kDa FITC-Dextran in the brain of Dhh^ECKO^ *vs* control mice has already been quantified and published by our laboratory and can be found in Figure 3, panel E of the Caradu C. et al. paper(2).

Additionally, we measured the distribution of 70 kDa FITC-Dextran within the gliovascular units of Dhh^ECKO^ and control mice by performing a LAM immunostaining on brain sections from control and Dhh^ECKO^ mice injected with 70 kDa FITC-Dextran (Fig S4, A-D).

The manuscript has been modified to annex these data (Line 307 to Line 321).

Figure 4

The conclusion that plasma leakage in Dhh KO mice is restricted by the glia limitans to the perivascular space is unfortunately not convincing. What is missing is a labelling with a pan laminin antibody to identify the endothelial and parenchymal basement membranes as described in Song et al, Cell reports 2015. Moreover, aquaporin 4 would have been a better marker of astrocytic endfeet. Indeed, as shown in panel D, GFAP labelling of astrocytic endfeet is inconstant. Also, podocalyxin, which is expressed on the luminal membrane of endothelial cells, cannot delineate the abluminal wall of the vessel.

We performed a co-immunostaining of PECAM1 (endothelial marker), AQP4 (astrocyte endfeet marker) and IgG (plasmatic protein extravasation marker) (Fig 4, A) and quantified the distribution of IgG in the 3 compartments (lumen, PVS and parenchyma) (Fig 4, B-C). The quantification protocol of IgG distribution within the lumen, PVS and parenchyma is highlighted in Fig S4, panel E.

Additionally, we did a co-immunostaining of LAM (endothelial and parenchymal basement membrane marker) and IgG to confirm the PVS trapping of plasmatic proteins in the Dhh^ECKO^ mice (Fig S5, A-B).

The manuscript has been modified to annex these data (Line 307 to Line 321).

The conclusion that "BBB breakdown is sufficient to induce a secondary CNS protective barrier at the Glia Limitans » is not supported by the data in the absence of the analysis of other mouse models with BBB leakage. This might depend on the cause of BBB leakage.

This sentence has been modified in the manuscript; we replaced it by the following sentence: « Dhh^ECKO^-induced BBB breakdown is sufficient to promote a secondary CNS protective barrier at the Glia Limitans » (Line 301 to Line 302) (Line 344 to Line 345)

However we have arguments in favor of a wider impact of BBB breakdown on the induction of astrocyte barrier properties. Indeed, we highlighted astrocyte CLDN4 expression in a model of BBB leakage induced by stereotactic injection of the pro-permeability factor VEGFA in the cortex of C57Bl/6 mice (Fig 5, K-O).

The manuscript has been modified to annex this information (Line 378 to Line 379).

Moreover, Glia Limitans barrier properties have also been identified and associated to BBB leakage in mouse models of neuro-inflammation (IL-1β stereotactic injection and EAE MOG_35-55_ models)(3).

The manuscript has been modified to annex this information (Line 532 to Line 534).

Another problem is that authors did not differentiate vessel types in their analysis. The concept of double-basement membrane structure of cerebral blood vessels makes sense in arteries/ arterioles or veins/venules but not in capillaries where the parenchymal and vascular basement membranes have merged.

In our study, we are focusing on arterioles and venules but not on capillaries.

This choice relies on a differential analysis of CLDN4 expression on neurovascular enriched fractions from Dhh^ECKO^ *vs* control mice. To perform this analysis, we added a filtration step just before harvesting the neurovascular enriched fractions for western blot quantification. 2 filters have been used, a 100 µm nylon mesh and a 20 µm nylon mesh. In both conditions, CLDN4 expression was quantified on the retained neurovascular enriched fractions. We highlighted that CLDN4 is only up regulated in Dhh^ECKO^ neurovascular enriched fractions which are 100µm and larger in diameter (Fig 4, F, H). In the samples obtained with neurovascular enriched fractions which are 20µm and larger, CLDN4 expression level was not different between the Dhh^ECKO^ and control group (Fig S6, B-D). In this condition, CLDN4 expression in arterioles and venules might have been lost in the crowd of capillaries.

The manuscript has been edited to include this information (Line 338 to Line 343).

Figure 5

Authors used VEGF or mannitol to simulate BBB leakage in vitro in cultured cells but there is no attempt to provide evidence that VEGF or mannitol did really something on their cells.

We performed a permeability assay *in vitro* to test the effect of PBS, VEGFA or D-Mannitol on HBMEC monolayer integrity. 150000 HBMECs were seeded in Transwell® inserts. The day after, PBS, VEGFA (10 ng/mL) (CliniSciences, Nanterre, France) or D-Mannitol (20 ng/µL) (Sigma Aldrich, Saint Louis, MI, USA) was added to the upper chamber for 6 hours. 0.5 mg/mL 70 kDa FITC-Dextran (Sigma Aldrich, St. Louis, MO, USA) was added to the upper chamber 5 hours and 30 min after treatment by the pro-permeability factors. FITC fluorescence in the lower chamber was measured 30 minutes later. We demonstrated that both VEGFA and D-Mannitol induce HBMECs monolayer permeability (Fig S7, A) (Line 377).

Again, there is a discrepancy between GFAP staining shown in panels E-H and GFAP quantification.

Representative images of GFAP expression in Figure 5, panels E to H have been replaced to make intensities of the GFAP labeling reflect the corresponding quantification data (Fig 5, I).

In the in vivo experiment the "hole drilled into the cerebral cortex", as mentioned in the material and methods, is likely to have induced unspecific astrocytic changes

This sentence has been modified in the manuscript; we replaced it by the following sentence: « Then, a hole was drilled into the skull, using a pneumatic station S001+TD783 Bien Air, until reaching the dura mater. 3μl of murine VEGFA (20 ng/µL), 3 µL of healthy mouse plasma or 3 µL of vehicle control (PBS) were then delivered at 0.01 μl/s into the frontal cortex at coordinates of 1 µm posterior to bregma, 2 µm left of the midline and 1.5 µm below the surface of the cortex (4). » (Line 749 to Line 753)

It is important to precise here that we are using a pneumatic station S001+TD783 Bien Air which allow us to be very gentle and precise when we do realize the drilling.

Figure 6

There seems to be a discrepancy between the alleged beneficial effect of inactivating Dhh on demyelination and the comparable lethality, unless the effect is marginal. Please explain.

The Y axis scale was wrong on the mortality rate graphic (Fig 6, D) and has been replaced. I apologize for this mistake. However, it doesn’t change the results interpretation.

The reduced clinical course of EAE in Dhh^ECKO^ mice is much more marked during the onset of the disease (between day 12 and day 20 post EAE induction). This is the result of increased astrocyte reactivity and implementation of barrier properties at the Glia Limitans in Dhh^ECKO^ mice, allowing for less diffusion of plasmatic proteins and immune cells into the CNS parenchyma than in control mice (Fig 6, A).

However, when the plateau phase is reached (after day 21 post EAE induction), the clinical score difference between the Dhh^ECKO^ and the control group is greatly reduced and coincides with an acceleration of the mortality rate in both groups (Fig 6, A, D).

One explanation to this observation is that, in the Dhh^ECKO^ group, inflammatory cells accumulated in the PVS end up degrading astrocytic tight junctions by secreting proteases. This phenomenon has already been observed and published(3) and explain that, after a while, inflammatory cells trapped in the PVS can eventually pass through the astrocytic barrier at the Glia Limitans and thereby enter the CNS.

Thus, we can conclude that inactivating Dhh at the BBB slow the disease progression until the perivascular accumulation of inflammatory cells causes the degradation of the astrocyte secondary barrier leading to the deterioration of the clinical course of EAE in the Dhh^ECKO^ mice (Fig 6, A). It explains the comparable lethality in both groups (Fig 6, D) but doesn’t take away the strong effect of Dhh^ECKO^ on disease progression during the onset phase of EAE (Fig 6, A, E).

The manuscript has been edited (Line 417 to Line 429) to clarify the results in Fig 6.

Figure 7

Panel E shows a diffuse astrocytic reaction in Dhh KO mice whereas fibrinogen staining seems to be restricted around what seems to be an arteriole. How do the authors reconcile this finding with their conclusion?

The diffuse astrocytic reaction within the CNS, following EAE MOG_35-55_ induction is a well-described phenomenon; astrocyte reactivity is induced, among others, by microglia *via* the secretion of inflammatory cytokines such as Il1α and TNF(5).

Here we show a diffuse astrocyte reaction in Dhh^ECKO^ mice but we also observe that this reaction is stronger at the Glia Limitans and associated to the perivascular entrapment of plasmatic proteins (Fig 7, C, E-F). In control mice, there is a diffuse astrocyte reaction in the EAE condition compared to the healthy condition (Fig 7, C). However there is no enhancement of astrocyte reactivity at the Glia Limitans (Fig 7, E-F). This is explained by the fact that, in Dhh^ECKO^ mice, astrocytic endfeet at the Glia Limitans are « preconditioned” to form a barrier which explains their ability, during neuropathology, to trap plasmatic proteins and inflammatory cells into the PVS to protect the parenchyma more efficiently than in the controls (Fig 7, E-J).

Also, in panel H vessels from the WT and Dhh KO mice are clearly different, with no perivascular cuff in the WT and a perivascular cuff in the KO mouse. Quantification of the number of CD45+ and T cells inside or outside this cuff is valid, provided that comparable vessels (arteries or veins) are analyzed in each genotype.

To better identify arteries and veins in panel H, we replaced the PDCXL/GFAP/CD45 labeling by a co-immunostaining of LAM and CD45 (Fig 7, G) and quantified the distribution of inflammatory cells between the PVS and the parenchyma specifically in arterioles (Fig 7, H).

The manuscript has been edited to annex these data (Line 460 to Line 470).

Others

In panel B (Fig 4) and in panels F-G, I-J (Fig 7) authors mention % values, but the confidence interval is not indicated. Also, it is very unclear how statistics have been performed. Comparison of % is usually analyzed by a Chi-square test but this is not mentioned in the Method section.

The statistical analysis of the distribution of plasmatic proteins (Fig 4, B-C) (Fig 7, F) and inflammatory cells (Fig 7, H) has been redone using a Chi-square test and the confidence intervals have been indicated.

The paragraph “statistical analysis” in the Method section has been updated (Line 829 to Line 830)

Reviewer #2: This paper by Hollier et al describes the functional emergence of a 2nd barrier formed by the glial limitans (astrocytes) following BBB breakdown in neuroinflammation. Molecularly, show that glial limitans 'tightening' to prevent parenchymal invasion of immune cells is a consequence of signals triggered by BBB breakdown (do this using a genetic model BBB breakdown, conditional deletion of Dhh and in MS animal model, also provide evidence in human MS tissue). This is very intriguing concept and builds off their 2017 JCI paper showing upregulation of astrocytic Cldn4 in MS animal models that helps to functional blocks parenchymal immune cell invasion that promotes disease progression. In general, I think this is a well-done study and of high interest to the fields of BBB mechanisms and neuroinflammatory disease. Below are my moderate concerns about some aspect of their analysis and a list of study limitations that should be addressed in the Discussion.

Major:

Line 115: Perivascular spaces are 'seen' around large penetrating arteriole and venules but I would check the literature carefully as I am not aware of the experimental evidence for PVS around capillary vasculature, which makes up the majority of vessels in the brain. At the capillary level, the astrocytes make direct and essentially continuous contact/coverage of abluminal surface. This becomes important later as the authors are proposing to analyze the PVS - laying out the literature of the existence of a space is important to support the validity of this analysis approach

The reviewer is absolutely right concerning the organization of the vascular tree in the CNS and the fact that there is no PVS identified to this day at the microvascular level. Thus has been very well described by C. Iadecola’s lab(6).

In our study, we are focusing on arterioles and venules but not on capillaries.

This choice relies on a differential analysis of CLDN4 expression on neurovascular enriched fractions from Dhh^ECKO^ *vs* control mice. To perform this analysis, we added a filtration step just before harvesting the neurovascular enriched fractions for western blot quantification. 2 filters have been used, a 100 µm nylon mesh and a 20 µm nylon mesh. In both conditions, CLDN4 expression was quantified on the retained neurovascular enriched fractions. We highlighted that CLDN4 is only expressed in Dhh^ECKO^ neurovascular enriched fractions which are 100µm and larger in diameter (Fig 4, F, H). In the samples obtained with neurovascular enriched fractions which are 20µm and larger, CLDN4 expression level was not different between the Dhh^ECKO^ and control group (Fig S6, B-D). In this condition, CLDN4 expression in arterioles and venules might have been lost in the crowd of capillaries.

The manuscript has been edited to include this information (Line 338 to Line 343).

Ln 191-193, Fig. 2: These are qualitative statements that should be supported by quantitative analysis of junctional disorganization - this data is similar to what was described in their 2018 CR paper, I would be satisfied if they stated this is consistent with previously documented phenotype using siRNA for Dhh (junctional disorganization was quantified in this previous paper and looks similar)

Mouse CNS MEC culture immunostaining were only used as a qualitative tool to show tight junction disorganization (Fig 2, D and F). As previously described by A.G Arroyo’s lab in 2014(1), what we were looking at is the breadth of CDH5 and CLDN5 at junctions. In controls, CDH5 and CLDN5 displayed a well-defined pattern of sharp contours at endothelial cell-cell contacts. By contrast, in Dhh^ECKO^ cultures, a broader, more irregular pattern was detected at endothelial cell-cell contacts (Fig 2, D and F). Because we have a broadened distribution and wider peak of CDH5 and CLDN5 intensity in Dhh^ECKO^ cells compared to controls doesn’t mean that their expression level is increased. On the contrary, it is a reflection of the endothelial CNS barrier dysfunction. This is why we are using qRT-PCR and western analysis as quantitative tools to measure CDH5 and CLDN5 expression levels in Dhh^ECKO^ and control CNS MECs (Fig 2, B and G).

The manuscript has been edited for clarity (Line 213 to Line 216).

The sentence “this result is consistent with previously documented phenotype using siRNA for Dhh(2)” has been added to the manuscript (Line 216 to Line 217).

Fig. 2: This group showed something similar in the 2018 paper Circulation Research paper, that paper also documented altered organization of Cdh5 at the junctions (with siRNA) but not a decrease in expression (but could be for experimental reasons) - the reduction of Cdh5 in these mutants is unclear, nor is the model of how Dhh is regulating this. For example they show Cdh5 down in their cultures, the mutants but why? This isn't discussed, just the decrease in Cldn5.

In our paper, we are studying blood vessels from the CNS in Dhh^ECKO^ mice *vs* control littermates whereas Caradu C. et al.(2) focused on the peripheral blood vessels in the same mouse model. Both vasculatures share many similarities notably in term of permeability and tight junction disorganization both *in vitro* and *in vivo*. However some markers react differently to the endothelial Dhh knockdown, particularly CDH5 expression level. Thus could be explained by the fact that we used human brain MECs and mouse CNS MECs to study CDH5 regulation *in vitro* whereas Caradu C. et al. used Human dermal MECs (HMVECs) and Human umbilical vein ECs (HUVECs). Endothelial cells at the BBB and blood spinal cord barrier display their own carrier, receptor and junction equipment; thus can be one explanation to the differential behavior of CDH5 expression to endothelial Dhh knockdown in both papers.

Concerning the mechanism underlying the down regulation of CDH5 expression in CNS MECs, one project in the lab is currently developed to respond to this specific question. We used BioGRID database to identify potential partners of the DHH receptor, PATCHED-1 (PTCH1). Among these we chose to test the role of Disintegrin and metalloproteinase domain-containing protein 17 (ADAM17), which implication in endothelial cell integrity has previously been described(7). Our data showed that treatment of ECs with soluble DHH induces ADAM17 protein degradation. To test whether DHH promotes adherens junction integrity by degrading ADAM17, ECs were then transfected with *Dhh* siRNA alone or with both *Dhh* and *Adam17* siRNAs. We found that while *Dhh* siRNA alone induces CDH5 junction destabilization and increased endothelial permeability, these effects were prevented in the presence of *Adam17* siRNA, demonstrating that *Dhh* knockdown promotes CDH5 junction destabilization and increased endothelial permeability via ADAM17.

Because this is an ongoing project and sensitive data, I won’t discuss it in the present study and hope the reviewer will understand this decision.

Fig. 3F: Please provide a low magnification image (can show current image as inset) to better demonstrate the scope of elevated ICAM expression. If the authors have this data, the ICAM with a blood vessel marker (PECAM) would help show this is vascular ICAM and how widespread it is in the spinal cord of the mutants.

We added lower magnification images which display co-immunostaining of ICAM1 and PECAM1 markers on spinal cord tissues from Dhh^ECKO^ mice and control littermates (Fig 3, G).

The manuscript has been edited to annex this information (Line 280 to Line 281)

Fig. 3G: Based on the image, it seems like the increase in GFAP is limited to the white matter, did quantification include all areas of spinal cord (grey and white)? This would be important to state in the methods and state if the increase was or was not regionalized (could do that by segregating/presenting the data by region).

We redid the quantification of GFAP expression on Dhh^ECKO^ *vs* control spinal cord tissues, presenting the quantification by region (white matter (WM) *vs* grey matter (GM)). Regionalization was highlighted by orange dotted lines on the images (Fig 3, H). As you can see, there is a strong up regulation of GFAP in the white matter (Fig 3, J) but no modulation of its expression in the grey matter (Fig 3, K).

The manuscript has been updated to take these observations into consideration (Line 281 to Line 283).

Fig. 4G - There is insufficient detail in the methods as to how this was quantified. Recommend showing higher magnification images, also highlighting using dotted lines outlines where measurements were taken (possibly a separate diagram or image with this information). In it's current form, it's very difficult for me to see how accurate this type of analysis is for differentiated between fibrinogen in and outside the vessel. Also, the 24% in the control is a little concerning, the expectation would this is zero, again leading to some concerns about this method. A limitation of this assay is the that fibrinogen is very large, possible there is a size restriction feature of the glial limitans barrier - (70kda FITC dextran appeared to leak well away from the vessel in the 2018 paper) - two options, perform experiments with a smaller size dye to test this or discuss this as a limitation of their findings in the Discussion (possibly small size proteins could leak through the

astrocytic barrier and still cause problems?)

To respond to a similar question from reviewer 1, we performed a co-immunostaining of PECAM1 (endothelial marker), AQP4 (astrocyte endfeet marker) and IgG (plasmatic protein extravasation marker) (Fig 4, A) and took pictures at a higher magnification to facilitate the quantification of IgG distribution between the 3 compartments (lumen, PVS and parenchyma). The quantification protocol of IgG distribution within the lumen, PVS and parenchyma is highlighted in Fig S4, panel E. We used negative working images of PECAM1-AQP4 channels and IgG channel. On the PECAM1-AQP4 images, we highlighted the endothelial and astrocyte basement membranes by using orange dotted lines and transferred these outlines to the IgG images so that we could quantify the distribution of IgG within the 3 compartments (lumen, PVS and parenchyma).

Using this markers and protocol, we redid the quantification of the repartition of plasmatic proteins within the neurovascular compartments in Dhh^ECKO^ and control mice and found that 5% of IgG is trapped in the PVS in the control mice (Fig 4, B-C) which is more relevant than the previous quantification number (24%), as the BBB is tight and functional in control mice. Additionally, we measured the distribution of 70 kDa FITC-Dextran within the neurovascular units of Dhh^ECKO^ and control mice by performing a LAM immunostaining on brain sections from control and Dhh^ECKO^ mice injected with 70 kDa FITC-Dextran (Fig S4, A). Using the same quantification protocol as above (Fig S4, B), we observed that 50,2% of 70 kDa FITC dextran is trapped in the PVS in the Dhh^ECKO^ mice against 2.9% in the control mice (Fig S4, C-D). Moreover, we didn’t observe any leak of 70 kDa FITC-Dextran in the parenchyma in the Dhh^ECKO^ mice (Fig S4, C-D).

These results confirm the effectiveness of the Glia Limitans in restraining access to the parenchyma to plasmatic proteins but also to a smaller size dye (Line 308).

The manuscript has been edited to annex these data (Line 309 to Line 321).

Ln 313-315 Fig. 5K-O: Need to show that VEGFA injection into the cortex causes BBB breakdown - or cite papers that have used this method before and shown this method is effective

VEGFA cortical stereotactic injection has been previously shown to efficiently induce BBB breakdown in mice(8,9).

This information has been added to the manuscript (Line 378 to Line 379)

Fig. 7 - this data is confusing as presented w/o the non-EAE data - to understand the effects of EAE model on the two genotypes, non-EAE values for each of these parameters should be included on the histograms - in comparing data from Fig 3 and Fig 7, a lot of the same measures were done - was the analysis done the same and therefore could it be added here and stats run to make comparisons?

Figure 7 panels A to D were modified; graphs now display FGB (Fig 7, A), CD45 (Fig 7, B), GFAP (Fig 7, C) and CDH5/IB4 (Fig 7, D) quantifications in control *vs* Dhh^ECKO^ mice under physiological condition and after EAE MOG_35-55_ induction. Thus, a comparison between physiological and pathological conditions can be done in control *vs* Dhh^ECKO^ animals as well as a comparison between the control and knockout animals in each condition.

The manuscript has been edited to annex these data (Line 451 to Line 453).

Ln 373: this is difficult to discern because Cdh5 expression is down, making it difficult to make out what is GFAP expression at glial limitans - remove statement or provide images/data that demonstrates this more clearly

Representative images of CDH5-GFAP-FGB immunostaining have been replaced to better illustrate the repartition of plasmatic proteins within neurovascular units in Dhh^ECKO^ and control mice (Fig 7, E). The CDH5 channel (grey) has been added to better discern the lumen area (Fig 7, E). The quantification protocol of FGB distribution within the lumen, PVS and parenchyma is highlighted in Fig S7, panel B. We used negative working images of CDH5-GFAP channels and FGB channel. On the CDH5-GFAP images, we highlighted the endothelial and astrocyte basement membranes by using orange dotted lines and transferred these outlines to the FGB images so that we can quantify the distribution of FGB within the 3 compartments (lumen, PVS and parenchyma).

The manuscript has been edited to annex these data (Line 454 to Line 459).

Fig. 7F, G: Similar comment to this type of analysis in Fig. 4A/B, need much better description in methods for how this was done, a diagram or annotated imaging showing where analysis was performed - while it's very clear from the images that fibrinogen is 'trapped' it's not clear how the quantitative data was acquired

To better identify arteries and veins in panel G, we replaced the PDCXL/GFAP/CD45 labeling by a co-immunostaining of LAM and CD45 (Fig 7, G) and quantified the distribution of inflammatory cells between the lumen, PVS and parenchyma (Fig 7, H).

The quantification protocol of CD45 distribution within the lumen, PVS and parenchyma is highlighted in Fig S7, panel C. We used negative working images of LAM and CD45 channel. On the LAM images, we highlighted the endothelial and astrocyte basement membranes by using blue dotted lines and transferred these outlines to the CD45 images so that we can quantify the distribution of leukocyte populations within the 3 compartments (lumen, PVS and parenchyma).

Using this markers and protocol, we redid the quantification of the repartition of leukocytes within the neurovascular compartments in Dhh^ECKO^ and control mice (Fig 7, H) and found a similar result to the one we previously obtained using the PDCXL/GFAP/CD45 labeling.

The manuscript has been edited to annex these data (Line 460 to Line 470).

Ln 410: A model would be very helpful to bring all the observations of this paper together

A scheme summarizing all the observations of our paper was added to the manuscript (Line 504)

Ln 429-431: I looked at Dhh and its expression in this dataset and it's actually very low vs other known endothelial genes - there could be MANY reasons for this, this was all from cortex, maybe there are regional differences, single cell can be tricky to pick up morphogens…I think the data in this manuscript is much more convincing of Dhh expression therefore I caution the authors against citing this as support for Dhh gene expression in brain endothelium

The reference from Christer Betsholtz’s lab(10) has been removed from the manuscript.

Ln 454-464 - One of the elements missing from this discussion is whether the increase in Cldn4 and astrocyte barrier induction is specific to the Dhh cKO model or is more broadly applicable to any mode of barrier opening - example in pericyte-deficient mice with BBB opening there is some problems with astrocyte-endfeet localization and the barrier opening is due to increased transcytosis no junctional degradation - bringing up these considerations is important

However, two questions remain regarding barrier properties at the Glia Limitans: (1) is this a specific aspect of the Dhh^ECKO^ mouse model or more broadly applicable to any model of BBB breakdown and (2) is it observed in other CNS pathologies than inflammation?

A model of pericyte deficient mice featuring BBB breakdown has been described; but, in this model, endothelial permeability is due to increased transcytosis and not junction degradation as in the Dhh^ECKO^ mouse model. Therefore it would be interesting to study the behavior of astrocytes in this model, notably their capacity to express CLDN4 and to restrain parenchymal access to plasmatic proteins. Should astrocyte barrier properties be observed in the pericyte deficient mouse model, thus would be suggestive for a more generalized role of BBB breakdown in driving astrocyte barrier properties, either by parallel or convergent mechanisms.

The question is now addressed in the discussion (Line 521 to Line 531)

Discussion: Another point that should be addressed in the discussion is whether this is a VEGFA driven process or broadly applicable to other BBB disrupters, example cytokines like IFNg - VEGFA but not mannitol was able to induce secretion of factors that signaled to astrocytes to increase Cldn4, what does this mean?

The expression of CLDN4 tight junction by reactive astrocytes has been demonstrated previously in a mouse model of cortical stereotactic injection of the pro-inflammatory cytokine IL-1β(3). This point has been added to the discussion (Line 532 to Line 534).

Osmotic agent D-Mannitol and pro-permeability factor VEGFA both induced HBMEC monolayer permeability (Fig S7, A) and D-Mannitol and VEGFA HBMECs conditioned media were both capable to up regulate GFAP (Fig 5, A, E-I) and CLDN4 expression (Fig 5, D, E-H, J) *in vitro*. We voluntary chose to only perform VEGFA cortical stereotactic injection *in vivo* because this model was already published(9,11) (Line 378 to Line 379) but also because the effect of D-Mannitol is very delimited in time (maximum effect observed between 2 and 8h post D-Mannitol treatment) which is too short to sacrificed the animals and harvest the brains after stereotactic surgery. In conclusion, both VEGFA and D-Mannitol were able to induce secretion of endothelial factors that signaled to astrocytes *in vitro* to induce CLDN4 expression (Fig 5, D, E-J) but because of experimental issue, we were not able to confirm the effect of D-Mannitol on CLDN4 astrocytic expression *in vivo*. However, based on the *in vitro* results, it seems reasonable to think that it will have the same effect as VEGFA.

Discussion: Another limitation to this study that should be discussed is it's not clear if this is a general function of Dhh to regulate the barrier or region specific, all analysis was done in the spinal cord.

The vascular extravasation of 70 kDa FITC-Dextran in the brain of Dhh^ECKO^ *vs* control mice has already been quantified and published by our laboratory and can be found in Fig 3, panel E of the Caradu C. et al. paper, 2018(2).

Further, it's also important to consider if the upregulation of astrocytic Cldn4 is limited to neuroinflammatory disease. This and their previous paper have only looked at EAE in spinal cord. What is the evidence that this could be MS specific phenomenon vs generalizable reaction to BBB opening in other context of other actute of chronic disease (stroke, TBI, AD, ALS, etc). For example, can point to their own data that VEGFA injection into the cortex induced Cldn4 upregulation in response to BBB disruptor VEGFA. Or have other studies looked at this Cldn4 upregulation in response to BBB opening?

The expression of CLDN4 by astrocytes has only been identified in mouse models of CNS inflammation (acute CNS inflammation model (stereotactic injection of the pro-inflammatory cytokine IL-1β and model of multiple sclerosis: EAE MOG_35-55_)(12). However, in the present study, we demonstrated that CLDN4 is expressed by astrocytes under resting conditions in response to BBB opening in the Dhh^ECKO^ mouse model and after cortical stereotactic injection of the pro-permeability factor VEGFA. The next step would be to examine astrocytic CLDN4 expression in other pathologies of the CNS, notably Alzheimer’s disease, Stroke or Amyotrophic Lateral Sclerosis in which BBB permeability has been identified as a critical pathophysiological player. This could implicate CLDN4-mediated barrier function in astrocytes as a more generalized defense against BBB opening in other chronic diseases of the CNS.

This question is now addressed in the discussion (Line 532 to Line 542).

Minor:

Ln 109: Edit sentence for clarity; also I would argue that this vision of the NVU has been building for years so suggest removing the term 'recent' and replace with language that reflects a wealth of literature that builds this idea of a multi-cellular 'BBB'.

The sentence has been edited for clarity and replaced in the manuscript by the sentence “Interestingly, a wealth of literature published during the last decades has enabled a change in the vision of BBB structure and integrity which has expanded to include contributions from both barrier properties of the vascular endothelial cells and the astrocytic endfeet of the neurovascular unit.” (Line 102 to line 105)

Ln 114: I would recommend adding a little more context for the reader - maybe a sentence saying this is a highly regulated process (solute transporters and receptor mediated trancytosis) and immune cells are actively prevented from cross by low levels immune receptors that normally permit immune trafficking.

We added a little more context for the reader by adding the following sentence to the manuscript: “Regulatory mechanisms at the BBB include solute transporters and receptor-mediated transcytosis, and immune cells are actively prevented from crossing the BBB by low levels of immune receptors that normally permit immune trafficking”. (Line 109 to line 112)

Ln 119: consider a different phrase, perhaps "is more complex"

The term “trickier” has been replaced in the manuscript by the words “is more complex”. (Line 117)

Ln 185: Please briefly describe how Dhh was deleted from ECs - are these ECs derived from the Cdh5-CreErtl Dhh1-fl/fl mice?

The sentence “To do so, we used isolated CNS MECs from *Cdh5-Cre^ERT2^;Dhh^Flox/Flox^* mice (Dhh^ECKO^ mice) and *Dhh^Flox/Flox^* control littermates, two weeks after inducing the knockdown by intra-peritoneal injection of tamoxifen.” has been added to the manuscript. (Line 205 to Line 208)

Ln 236: edit "BBB" to say brain endothelium since ICAM is expressed by the endothelial cells not the BBB per se.

The term “BBB” has been replaced in the manuscript by the term “spinal cord endothelium”. (Line 275)

Ln 271: "human disease" instead of "Human"

The term “Human” has been replaced in the manuscript by the term “human disease”. (Line 324)

Ln 281: typo?

We couldn’t find any typo in the manuscript

Ln 420: edit "proved" to say "showed"

The term “proved” has been replaced in the manuscript by the term “showed”. (Line 515)

Ln 420-424: break up in to two sentences

The sentence has been broke up in two: “Moreover, we showed that in Dhh^ECKO^ mice, which display an open BBB, astrocytes express the tight junction protein CLDN4 under resting conditions. Therefore, under neuro-inflammatory conditions, Glia Limitans in Dhh^ECKO^ mice is primed with stronger barrier properties protecting against the onset and severity of EAE symptoms in the Dhh^ECKO^ mice compared to control littermates”. (Line 515 to Line 520	)

Reviewer #3: In the present study Chapouly and her team provide convincing evidence for an additional role of the glia limitans in regulating CNS immunity, when BBB integrity is impaired. They have focused on the role of Dhh and show when deleted from the endothelium in mice, BBB tightness is impaired and at the same time tightness of the glia limitans is increased possibly by the induction of tight junctions in astrocytes. DhhECKO mice develop delayed EAE with CD45+ cells found to be trapped in perivascular spaces underscoring the notion of an increased barrier property of the glia limitans in these mice.

The study combines analysis of human and mouse tissues and the finding of the cross-talk of an impaired BBB leading to improved barrier properties of the glia limitans to immune components is quite novel.

The study as it stands has however a significant number of issues that need to be addressed. Overall the authors seem to tend towards conclusions that are not yet supported by the data as presented. Thus overall downtoning of the manuscript will be required.

I am absolutely aware that in the COVID-19 pandemic a number of issues may not be solvable as they require additional experiments. If additional data cannot be provided the authors need to explain the reason and instead significantly downtone their conclusions.

The authors base their study on the statement that "Presently, it is accepted that the BBB is the sole line of defense of the CNS, restricting access to the parenchyma to inflammatory infiltration notably in the context of chronic neuroinflammation." This is certainly not the case and the authors should include mentioning and discussion of the work of e.g. the Sorokin laboratory, which has provided mechanistic insight into the role of the glia limitans in CNS inflammation. Also Joan A Abbott has written several articles highlighting the role of the glia limitans as a brain barrier. In numerous reviews including those of Weller, Owens, Ransohoff and Engelhardt data on the role of the the glia limitans has been highlighted as barrier for the immune system.

The statement "Presently, it is accepted that the BBB is the sole line of defense of the CNS, restricting access to the parenchyma to inflammatory infiltration notably in the context of chronic neuroinflammation." has been nuanced and replaced in the manuscript by the sentence “While it is now well established that BBB breakdown leads to soluble factor and inflammatory cell infiltration into the PVS during neuropathology^4^, the Glia Limitans barrier function is just starting to be unraveled(12–15)” (Line 506 to Line 508).

Additionally a discussion introducing the work of the laboratories that identified astrocyte barrier properties has been added to the manuscript: “Astrocyte barrier properties are not as well characterized as those of the BBB. However, several groups have highlighted barrier properties at the Glia Limitans(14,16,17). Notably, endfoot-endfoot clefts, similar to those observed between endothelial cells at the BBB, have been described at the Glia Limitans and shown to be responsible for the sieving effect observed between the distribution of small and large Dextrans(18). Moreover, under neuro-inflammatory conditions, immune cell trafficking across the Glia Limitans is necessary for clinical EAE(16); indeed, matrix metalloproteinase (MMP)-2 and MMP-9 proteolytically cleave dystroglycan, which anchors astrocyte endfeet to the Glia Limitans basement membrane *via* binding to extracellular matrix molecules, allowing infiltrating leukocytes to penetrate the parenchyma(15). Additionally, it has been shown that the scavenger receptor CXCR7 is upregulated on the inflamed BBB endothelium, facilitating mobilization of T cells from the PVS into the CNS parenchyma(19). Altogether, these data demonstrate that both the endothelial BBB and its basement membrane, along with the Glia Limitans and the parenchymal basement membrane, are required for immune cell trafficking across the neurovascular unit. Strikingly, our recent work has given considerable attention to a new property of reactive astrocytes: the expression of tight junction proteins (notably Claudin4 (CLDN4)) under inflammatory conditions(12). This result provides yet another argument in favor of astrocytic barrier properties.” (Line 124 to Line 141)

Throughout the manuscript the authors make repeatedly statements as the following that need to be corrected as suggested:

*	that the adherens junction Cdh5 and tight junction Cldn5 are down regulated

o	downregulation of mRNA of junctional molecules does not allow to make any statement on the integrity or overall molecular composition of a cellular junction

The sentence “adherens junction Cdh5 and Tight junction Cldn5 are downregulated” has been replaced throughout the manuscript by the following sentences:

- “with down regulation of mRNA markers of tight junctions (*Cldn5* and *Zo1*)” (Line 178 to Line 179)
- “to maintain endothelial adherens and tight junction mRNA and protein expression level at the BBB and to maintain a well-defined CDH5, ZO1 and CLDN5 pattern of sharp contours at endothelial cell-cell contacts.” (Line 219 to Line 221)

*	Isolation of neurovascular unit

o	This unit of cellular and acellular components can hardly be isolated

The term “neurovascular unit” has been replaced by the term “neurovascular fraction enrichment” throughout the manuscript.

*	Gliovascular unit

o	what do the authors mean with this expression as compared to neurovascular unit?

The term “gliovascular unit” has been replaced by the term “neurovascular unit” throughout the manuscript.

o	a network of astrocytes termed the Glia Limitans.

o	The glia limitans is composed of the parenchymal basement membrane and the astrocyte end-feet rather than a network of astrocytes.

This sentence has been rephrased and replaced in the manuscript by the sentence “Once soluble factors and immune cells penetrate the BBB, they circulate within the perivascular space (PVS), a region between the basal basement membrane of the endothelial cell wall and the parenchymal basement membrane abutting the astrocyte endfeet(13,20).” (Line 112 to Line 115).

Endothelial adherens and tight junction expression is maintained in DhhECKO

This could only be stated when functional and ultrastructural studies confirm this.

A panel has been added to the Fig 2 showing that in Dhh^ECKO^ CNS MEC cultures treated with the inhibitor of FOXO1, CLDN5 displays a well-defined pattern of sharp contours at endothelial cell-cell contacts. By contrast, in Dhh^ECKO^ CNS MEC cultures treated with DMSO, a broader, more irregular pattern is detected at endothelial cell-cell contacts (Fig 2, M).

Moreover, the sentence “Endothelial adherens and tight junction expression is maintained in Dhh^ECKO^” has been replaced in the manuscript by the following sentence: “We concluded that endothelial DHH expression is necessary to maintain endothelial adherens and tight junction mRNA and protein expression level at the BBB and to maintain a well-defined CDH5, ZO1 and CLDN5 pattern of sharp contours at endothelial cell-cell contacts.” (Line 219 to Line 222)

Comments to the data in the sequence of the manuscript:

In general the authors refer to the gene names when staining or detecting proteins in IF stainings or WB -this is not correct and needs to be corrected.

The gene (mouse: italic lowercase letters, human: italic capital letters) and protein (mouse and human: capital letters) nomenclatures have been corrected throughout the manuscript.

Figure 1 A shows lack of expression of Shh and Ihh in the healthy brain of mice - this is in apparent contrast to the observations by the Prat lab that has identified astrocytes as source of Shh maintining BBB stability, which is mentioned by the authors. The authors should compare the primer sets used and either repeat the assay or refer to the apparent difference.

In Fig 1 panel A, we quantified the expression level of *Shh* and *Ihh* mRNAs in primary CNS MEC cultures (7 days post seeding).

CNS MEC culture purity was assessed by quantifying *Pecam1* mRNA expression level and *Sma*, *Ng2*, *Cd45* and *Gfap* mRNA expression level to assess smooth muscle cell, pericyte, leukocyte and astrocyte contaminations respectively (Fig 1, A). Additionally, we performed immunostaining on primary CNS MEC cultures using ZO1 to identify the endothelial cell layer and NG2, SMA, GFAP and IBA1 to highlight pericyte, smooth muscle cell, astrocyte and microglia contaminations (Fig S1, A). As expected *Pecam1* and ZO1 expressions are predominant in the CNS MEC preparations but there is a low contamination by other components of the neurovascular unit especially by pericytes and smooth muscle cells. However, astrocyte contamination is very low (Fig 1, A) (Fig S1, A) which explain why *Shh* expression can’t be detected by qRT-PCR in primary CNS MEC cultures.

The manuscript has been edited to annex this information (Line 163 to Line 166).

Figure 1 B does not show any overlap of VE-cadherin staining with the Dhh staining, nevertheless the authors conclude that Dhh is expressed in CNS endothelium.

There is overlapping areas of CDH5 and DHH staining in Fig 1 panel B. We added an enlarged window of one of the overlap to better highlight the CDH5 and DHH merged signals (Fig 1, B).

Figure 1F to J: Isolation of spinal cord microvessels from mice suffering from EAE will contain other contaminants as microvessels isolated from healthy controls. Lower levels of Dhh, Claudin-5 and ZO-1 from the vascular compartment accompanied by higher levels of ICAM-1, VCAM-1 could also be obtained by coisolation of a higher number of contaminations with astrocyte end-feet, perivascular inflammatory cells or their debris or regulated expression in pericytes and smooth muscle cells.

We thank the reviewer for this very useful comment. We quantified the expression level of *Cd45*, *Sma*, *Ng2* and *Gfap* by qRT-PCR in the control *vs* Dhh^ECKO^ isolated micro-vessels to measure their contamination by leukocytes, smooth muscle cells, pericytes and astrocytes. If the contamination by smooth muscle cell, pericyte and astrocyte is low and similar in both groups, the contamination by leukocytes is strongly increased in the Dhh^ECKO^ mice compared to control littermates (Fig S1, D). Therefore, we redid the experiment; this time, C57Bl/6 mice were induced with EAE MOG_35-55_ *vs* placebo (Fig 1, F-J) and at day 13 post induction (we used an earlier stage of disease progression to respond to one question from reviewer 1), spinal cord micro-vessels were isolated (using a gradient of Dextran) and underwent a digestion step followed by a CD45^+^ T cell depletion step using a MACS CD45 T cell isolation kit (Miltenyi Biotec) to discard the contamination by inflammatory cells induced by EAE (Fig S1, C-D). The CD45^+^ T cell depletion step efficiency was confirmed by flow cytometry on 3 Dhh^ECKO^ vs 3 control samples (Fig S1, C).

Lower levels of *Dhh*, *Cldn5* and *Zo1* from the vascular compartment accompanied by higher levels of *Icam1* and *Vcam1* were finally validated in the new samples (Fig 1, F-J).

The manuscript has been edited to annex these data (Line 174 to Line 177).

In this context the author refer to their results in Fig 1, I-J showing lower levels of mRNA for claudin-5 and ZO1, as "down regulation of tight junctions"- this is inappropriate as tight junctions are complex cell-cell-contacts including more than describing the mRNA expression of their proteins.

The sentence « down regulation of tight junctions » has been replaced by the sentence «down regulation of the mRNA expression level of *Cldn5* and *Zo1*” throughout the manuscript.

Additionally, in Fig 7 panel D, CDH5 protein expression level has been quantified on spinal cord sections from Dhh^ECKO^ vs control mice under resting condition and after EAE MOG_35-55_ induction. We observed that CDH5 protein expression level is down regulated in Dhh^ECKO^ mice under resting condition and in both Dhh^ECKO^ and control mice after EAE induction. This result is a further argument in favor of a destabilization of BBB endothelial junctions during EAE progression at a protein level.

Figure 2A - The authors verify endothelial cell specific deletion of Dhh in vivo by determining mRNA levels for Dhh in endothelial cell cultures derived from these mice. This does of course not allow to verify the efficiency of Dhh depletion in vivo as it does now allow to determine if Dhh has been deleted with different efficiency from different parts of the CNS vasculature.

To verify the efficiency of Dhh depletion *in vivo* in the Dhh^ECKO^ mouse model in different parts of the CNS vasculature, *Cdh5-Cre^ERT2^* mice were bred together with *Rosa26^mTmG^* mice. Mice were sacrificed 2 weeks, 4 weeks, 6 months and 1 year after they were administered with tamoxifen. Brain and spinal cord tissues were harvested and cross sections were immunostained with anti-GFP and anti-PECAM1 antibodies to identify cells in which recombination occurred. We measured recombination efficacy in cerebellar, cortical, corpus callosum and spinal cord cross sections for the 4 weeks post tamoxifen time point (Fig S2, A); 100% of blood vessels expressed GFP which means that *Cdh5-Cre^ERT2^* efficiency and therefore Dhh knockdown is robust in various parts of the CNS.

The manuscript has been edited to annex this information (Line 631 to Line 633).

It could thus be that the authors culture endothelial cells from different parts of the vascular tree as they also don't know if lack of Dhh has any effect in survival of the ECs during isolation or their growth in vitro.

To measure any effect of Dhh knockdown on CNS MEC survival, we cultured Dhh^ECKO^ *vs* control CNS MECs from a pooled source including both brain and spinal cord tissues. 7 days post seeding, we measured cell viability in the Dhh^ECKO^ *vs* control CNS MEC cultures using propidium iodide treatment (Fig S1, E-F). No difference was observed between the Dhh^ECKO^ and the control CNS MEC cultures in term of cell viability with less than 4% of endothelial cell propidium iodide incorporation in both groups (Fig S1, E-F).

The manuscript has been edited to annex this information. (Line 208 to Line 209)

Furthermore, considering that Dhh is a morphogen it is mandatory to investigate how Cre-mediated deletion of Dhh affects brain angiogenesis.

Brain and spinal cord angiogenesis were quantified using IB4 labeling of Dhh^ECKO^ and control mouse brain and spinal cord cross sections. The number of vessels wasn’t different in the brain and spinal cord of Dhh^ECKO^ and control mice (Fig S2, B-C).

The manuscript has been edited to annex this information (Line 632 to Line 633).

Figure 3: The authors refer to ICAM-1 and GFAP as markers analyzing the "activation status of both the BBB and Glia Limitans". As outlined several times using a reference molecule to describe an entire tissue or cell is not appropriate. Although ICAM-1 is an excellent marker for EC activation, in the context of the inflamed CNS tissue the authors have to consider that ICAM-1 is also upregulated on pericytes, smooth muscle cells and astrocytes.

In Fig 3, we are not in CNS inflamed tissues; we are analyzing spinal cord cross sections from Dhh^ECKO^ and control mice under resting condition, measuring the effect of Dhh endothelial knockdown on vessel and astrocyte reactive behavior (Fig 3, G-K). We chose ICAM1 as a marker of endothelial activation (Fig 3, G, I) and GFAP as a marker of astrocyte reactivity (Fig 3, H, J-K) because they are both widely published in the context of inflamed CNS tissues but we do realize that using only one reference molecule does not allow us to fully characterize the activation status of endothelial cells and astrocytes in the Dhh^ECKO^ vs control mice. It only represents one strong indicator of a reactive response of the BBB and Glia Limitans to Dhh endothelial knockdown.

The manuscript has been edited to annex this information (Line 276 to Line 279).

Additionally, we added lower magnification images which display co-immunostaining of ICAM1 and PECAM1 markers on spinal cord tissues from Dhh^ECKO^ mice and control littermates to confirm that ICAM1 is expressed by endothelial cells and not by pericytes, smooth muscle cell or astrocytes (Fig 3, G) (Line 280 to Line 281).

Figure 3A - why do the authors show two sets for VE-cadherin/Fibrinogen staining? It would be preferable to show another set e.g. for claudin-5 and albumin staining.

In Fig 3, one set for CDH5/FGB staining has been removed and replaced by a set for CDH5/IB4 staining to quantify CDH5 area over the vessel area, to normalize for the number and length of vessels. Additionally, in Fig S3, CLDN5/IB4/DAPI (Figure S3, A) and CLDN5/ALB (Fig S3, C) staining have been added.

Figure 4B: The authors have used podocalyxin and GFAP to define localization of fibrinogen in the lumen, perivascular space and within the CNS parenchyma. The perivascular space is bordered by basement membranes, which can be detected by pan-laminin stainings which would be preferable for accurate analysis of the localization of fibrinogen. The authors thus either perform additional stainings or significantly downtone this paragraph as GFAP as an intermediate filament, which is also not detectable in all astrocytes does not allow for accurate definition of the glia limitans.

To respond to a similar question from reviewer 1, we performed a co-immunostaining of PECAM1 (endothelial marker), AQP4 (astrocyte endfeet marker) and IgG (plasmatic protein extravasation marker) (Fig 4, A) and quantified the distribution of IgG in the 3 compartments (lumen, PVS and parenchyma) (Fig 4, B-C). The quantification protocol of IgG distribution within the lumen, PVS and parenchyma is highlighted in Fig S4, panel E.

Additionally, we did a co-immunostaining of LAM (endothelial and parenchymal basement membrane marker) and IgG to confirm the PVS trapping of plasmatic proteins in the Dhh^ECKO^ mice (Fig S5, A-B).

The manuscript has been edited to annex these data (Line 307 to Line 321).

Claudin-4 is suggested to be expressed in GFAP + astrocytes- the staining for both markers looks to a large degree identical, which is confusing as GFAP is an intermediate filament whereas claudin-4 would be expected at the cell membranes and possibly focused to astrocyte end feet. Did the authors check for other claudins and could confirm specificity of this staining?

We performed CLDN5/GFAP immunostaining on active cortical lesions from multiple sclerosis patients; there was absolutely no overlap of CLDN5 and GFAP signals (see attached). Both anti-CLDN4 (mouse) and anti-CLDN5 (mouse) antibodies are from Invitrogen (Carlsbad, CA, USA) and used at the same dilution.

**CLDN-4 is upregulated in a mouse model of CNS inflammatory disease:** Spinal cord sections were harvested from C57Bl/6 and immunostained with anti-GFAP, anti-CLDN-4, anti-Laminin antibodies. Nuclei were stained with DAPI (in blue). Scale bar 15µm

Human active multiple sclerosis cortical lesions were obtain from the NeuroCEB biobank and immunostained with anti-CLDN5 (in green) and anti-GFAP (in red) antibodies. Nuclei were stained with DAPI (in blue).

Additionally, we did observed the same labeling pattern of CLDN4 in GFAP+ astrocytes in a mouse model of EAE MOG_35-55_ (see attached)(9).

CLDN4 labeling intensity is stronger at the Glia Limitans on active cortical lesions from multiple sclerosis patients as indicated by white arrows in Fig 4, panel D even if we do observe CLDN4 labeling in astrocytes not in the immediate vicinity of a blood vessel (Fig 4, D).

The manuscript has been edited to annex this information (Line 324 to Line 326).

Figure 4 E: WB signal for claudin-4 looks very weak. A positive control with claudin-4 transfectants would be helpful to show.

Western blot analysis of CLDN4 expression was performed by loading Dhh^ECKO^ mouse neurovascular enriched fractions and C57Bl/6 mouse small intestine lysates as a positive control(21,22) (Fig S6, A).

The manuscript has been edited to annex this information (Line 331 to Line 332).

Figure 5: The authors delivered VEGF-A or I guess what they mean plasma proteins into the left cerebral cortex of adult mice. They have used a very high volume of 3 μL which in addition was injected in only 30 seconds !!!! into the tissue - this will cause massive damage of the tissue, which needs to be considered when interpreting these data.

There is a mistake in the paragraph “Stereotactic injection” of the Method section. We wrote “3μl of murine VEGFA (20 ng/µL), 3 µL of healthy mouse plasma or 3 µL of vehicle control (PBS) were delivered at 0.1 μl/s into the frontal cortex”. We delivered the VEGFA, plasmatic proteins or vehicle control at 0,01 µl/s. We apologize for this mistake. The sentence has been replaced in the manuscript by the following sentence: “3μl of murine VegfA (20 ng/µL), 3 µL of healthy mouse plasma or 3 µL of vehicle control (PBS) were delivered at 0.01 μl/s into the frontal cortex” (Line 750 to Line 753).

Concerning the volume of 3 µL, we already published this protocol several times before(8,9) and it has been approved by the local Animal Care and Use Committee of the Bordeaux University CEEA50 (IACUC protocol #16901). Additionally, by using PBS as a vehicle control, we can control that we don’t have any cortical tissue damage at the site of injection.

Figure 6: The authors show delayed onset of EAE in the DhhEC KO mice. As mentioned above without prior analysis of the impact of endothelial cell specific deletion of Dhh in the entire vasculature of these mice, conclusions from these data are difficult to make.

CNS angioarchitecture is the minimum that needs to be studied, but vascular deletion of Dhh could have effects beyond the CNS not considered by the authors but still impacting on EAE which is induced by peripheral activation of T cells that need to travel to the CNS.

Endothelial specific deletion of Dhh has been analyzed in the peripheral vasculature in a paper from our group published in 2018(2). In this paper, we showed that endothelial specific deletion of Dhh in the peripheral vasculature is associated with vascular permeability and endothelial activation, notably in the lung, and that LPS injection increased pulmonary neutrophil infiltration in Dhh^ECKO^ mice compared to control littermates. Therefore, we can hypothesize that endothelial Dhh knockdown increases the peripheral recruitment and activation of inflammatory cells that need to travel to the CNS. However, in our study, we do not observe any difference in terms of CD45+ leukocyte populations in the spinal cord of Dhh^ECKO^ and control mice induced with EAE MOG_35-55_. What we do observe is a significant difference in terms of the repartition of these CD45+ cell populations between both groups, with 77.1 % of CD45+ cells trapped into the PVS in the Dhh^ECKO^ cohort versus 25.1 % in the control cohort. The manuscript has been edited to annex these data (Line 460 to Line 470).

To analyze Dhh^ECKO^ and control mouse CNS angio-architecture, we performed microcomputed tomography vasculature imaging. 3 Dhh^ECKO^ mice and 3 control littermates were perfused with a mixture containing 80% neoprene latex (Neoprene Latex Dispersion 671 A; DuPont, France) and barium sulfate powdered to 1 μm (3 g/mL, Micropaque oral solution, Guerbet, France). Brain vasculature was imaged using a high-resolution microcomputed tomography imaging system (eXplore Locus SP; General Electric Healthcare). Image acquisitions and reconstructions were performed using Scan Control and Reconstruction Utility programs. After analysis of all the vascular tree reconstructions, we couldn’t see any difference between the Dhh^ECKO^ and control brain angio-architecture (Fig S2, D).

The manuscript has been edited to annex this information (Line 632 to Line 633).

Last but not least the manuscript does need editing with the help of a native speaker.

The manuscript has been edited with the help of a native speaker.

Bibliography

1.	Moreno V, Gonzalo P, Gomez-Escudero J, Pollan A, Acin-Perez R, Breckenridge M, et al. An EMMPRIN- -catenin-Nm23 complex drives ATP production and actomyosin contractility at endothelial junctions. J Cell Sci. 1 sept 2014;127(17):3768‑81.

2.	Caradu C, Couffinhal T, Chapouly C, Guimbal S, Hollier P-L, Ducasse E, et al. Restoring Endothelial Function by Targeting Desert Hedgehog Downstream of Klf2 Improves Critical Limb Ischemia in Adults. Circ Res. 12 2018;123(9):1053‑65.

3.	Horng S, Therattil A, Moyon S, Gordon A, Kim K, Argaw AT, et al. Astrocytic tight junctions control inflammatory CNS lesion pathogenesis. J Clin Invest. 1 août 2017;127(8):3136‑51.

4.	Argaw AT, Gurfein BT, Zhang Y, Zameer A, John GR. VEGF-mediated disruption of endothelial CLN-5 promotes blood-brain barrier breakdown. Proc Natl Acad Sci U S A. 10 févr 2009;106(6):1977‑82.

5.	Liddelow SA, Guttenplan KA, Clarke LE, Bennett FC, Bohlen CJ, Schirmer L, et al. Neurotoxic reactive astrocytes are induced by activated microglia. Nature. janv 2017;541(7638):481‑7.

6.	Iadecola C. The Neurovascular Unit Coming of Age: A Journey through Neurovascular Coupling in Health and Disease. Neuron. 27 sept 2017;96(1):17‑42.

7.	Shen M, Hu M, Fedak PWM, Oudit GY, Kassiri Z. Cell-Specific Functions of ADAM17 Regulate the Progression of Thoracic Aortic Aneurysm. Circ Res. 20 juill 2018;123(3):372‑88.

8.	Argaw AT, Zhang Y, Snyder BJ, Zhao M-L, Kopp N, Lee SC, et al. IL-1β Regulates Blood-Brain Barrier Permeability via Reactivation of the Hypoxia-Angiogenesis Program. J Immunol. 15 oct 2006;177(8):5574‑84.

9.	Chapouly C, Tadesse Argaw A, Horng S, Castro K, Zhang J, Asp L, et al. Astrocytic TYMP and VEGFA drive blood-brain barrier opening in inflammatory central nervous system lesions. Brain J Neurol. juin 2015;138(Pt 6):1548‑67.

10.	He L, Vanlandewijck M, Mäe MA, Andrae J, Ando K, Del Gaudio F, et al. Single-cell RNA sequencing of mouse brain and lung vascular and vessel-associated cell types. Sci Data. 21 2018;5:180160.

11.	Argaw AT, Asp L, Zhang J, Navrazhina K, Pham T, Mariani JN, et al. Astrocyte-derived VEGF-A drives blood-brain barrier disruption in CNS inflammatory disease. J Clin Invest. juill 2012;122(7):2454‑68.

12.	Horng S, Therattil A, Moyon S, Gordon A, Kim K, Argaw AT, et al. Astrocytic tight junctions control inflammatory CNS lesion pathogenesis. J Clin Invest. 1 août 2017;127(8):3136‑51.

13.	Engelhardt B, Ransohoff RM. Capture, crawl, cross: the T cell code to breach the blood-brain barriers. Trends Immunol. déc 2012;33(12):579‑89.

14.	Engelhardt B, Carare RO, Bechmann I, Flügel A, Laman JD, Weller RO. Vascular, glial, and lymphatic immune gateways of the central nervous system. Acta Neuropathol (Berl). 2016;132(3):317‑38.

15.	Song J, Wu C, Korpos E, Zhang X, Agrawal SM, Wang Y, et al. Focal MMP-2 and MMP-9 Activity at the Blood-Brain Barrier Promotes Chemokine-Induced Leukocyte Migration. Cell Rep. 24 févr 2015;10(7):1040‑54.

16.	Di Russo J, Hannocks M-J, Luik A-L, Song J, Zhang X, Yousif L, et al. Vascular laminins in physiology and pathology. Matrix Biol. 1 janv 2017;57‑58:140‑8.

17.	Abbott NJ, Rönnbäck L, Hansson E. Astrocyte-endothelial interactions at the blood-brain barrier. Nat Rev Neurosci. janv 2006;7(1):41‑53.

18.	Iliff JJ, Wang M, Liao Y, Plogg BA, Peng W, Gundersen GA, et al. A Paravascular Pathway Facilitates CSF Flow Through the Brain Parenchyma and the Clearance of Interstitial Solutes, Including Amyloid β. Sci Transl Med. 15 août 2012;4(147):147ra111-147ra111.

19.	Cruz-Orengo L, Holman DW, Dorsey D, Zhou L, Zhang P, Wright M, et al. CXCR7 influences leukocyte entry into the CNS parenchyma by controlling abluminal CXCL12 abundance during autoimmunity. J Exp Med. 14 févr 2011;208(2):327‑39.

20.	Engelhardt B, Coisne C. Fluids and barriers of the CNS establish immune privilege by confining immune surveillance to a two-walled castle moat surrounding the CNS castle. Fluids Barriers CNS. 18 janv 2011;8(1):4.

21.	Nagatake T, Fujita H, Minato N, Hamazaki Y. Enteroendocrine Cells Are Specifically Marked by Cell Surface Expression of Claudin-4 in Mouse Small Intestine. PLOS ONE. 6 mars 2014;9(3):e90638.

22.	Troeger H, Loddenkemper C, Schneider T, Schreier E, Epple H-J, Zeitz M, et al. Structural and functional changes of the duodenum in human norovirus infection. Gut. 1 août 2009;58(8):1070‑7.

__________________________________________________

In compliance with data protection regulations, you may request that we remove your personal registration details at any time. (Use the following URL: <https://www.editorialmanager.com/pbiology/login.asp?a=r>). Please contact the publication office if you have any questions.
