# Supplementary material for: Blood–brain barrier genetic disruption leads to protective barrier formation at the Glia Limitans
Source: PLoS Biol. 2020 Nov 30;18(11):e3000946. doi: 10.1371/journal.pbio.3000946 (PMC7728400; doi:10.1371/journal.pbio.3000946)
Supplement: Attachment — Submitted filename: Response to reviewers.docx [file pbio.3000946.s011.docx]

**Response to reviewers**

*Reviewer #1: In their revised manuscript, Mora et al., have added new data and clarified some issues. However, important concerns remain to be resolved.*

*In figure 2, the finding that CLDN5 is downregulated in MEC from Dhh KO mice is still not convincing. Contrary to what is mentioned in the rebuttal, expression of CLDN5 has not been analyzed by immunoblot and the panel F clearly shows an upregulation of CLDN5.*

**Cldn5 expression is downregulated in DhhECKO CNS MECs:** CNS MECs were isolated from Dhh^ECKO^ and control mice, seeded and cultured until confluency and Cldn5 expression was quantified by western blot.

The expression level of Cldn5 in Dhh^ECKO^ CNS MECs has been analyzed and quantified by western blot in another research article from our group which has just been accepted for publication in Cardiovascular Research. You can find enclosed the panel showing the down regulation of Cldn5 protein expression in isolated Dhh^ECKO^ CNS MECs by western blot.

*The conclusion that plasma leakage in Dhh KO mice is restricted by the glia limitans to the PVS is still not convincing. 1) In figure 4, panel A, there is clearly an uptake of IgG in the astrocyte labeled by the anti-aquaporin 4 antibody.*

We agree with Reviewer #1 that there is some overlap between the IgG signal (green) and the AQP4 signal (red) in Fig 4 panel A. However, the uptake of IgG, in the astrocytes labeled by the anti-AQP4 antibody, is concentrated within the internal surface of the Glia Limitans. It is actually an interesting observation as it might reflect a potential interaction between the inner face of astrocyte endfeet and IgG accumulated in the PVS. Indeed, astrocytes express Fc Receptors (cell surface receptors for IgG) and the literature showed that Fc Receptors play a role in the CNS health and disease(1,2).

The manuscript has been edited (Line 289 to Line 294) to clarify the results in Fig 4.

*2°) If the glia limitans restricts plasma leakage in DhhKO mice, then fibrinogen staining in Figure 3 panel shall be exclusively detected in the PVS around arteries or veins but not around capillaries, where there is a single composite basement membrane produced by endothelial cells, pericytes and astrocytic and no PVS. This does not seem to be the case in this figure 3, quality of which is quite poor.*

We did not explore the distribution of plasmatic proteins at the capillary level in the Dhh^ECKO^ mouse CNS. This is why we are only concluding on the BBB status in Fig 3 (Fig 3, A-F) as stated in the manuscript: “*In vivo*, on spinal cord sections from control and Dhh^ECKO^ mice, we confirmed that the adherens junction CDH5 and tight junction CLDN5 expression, when normalized for the number and length of blood vessels, are down regulated under resting conditions (Fig 3, A-D) (S1 Data), (S3 Fig, A) and demonstrated that it is associated to an increase accumulation of serum proteins (FGB and ALB)(3) (Fig 3, B, E-F) (S1 Data), (S3 Fig, B-C) around the vessels suggesting BBB opening.” (Line 237 to Line 242)

The manuscript has been edited to clarify the fact that we are only quantifying the distribution of plasmatic proteins in arterioles and venules. (Line 276 to Line 277)

We had to reduce the large file size to proceed to the submission of the manuscript which explains the quality loss of the immunofluorescence image (Fig 3, B). The high quality image is available and can be sent to Reviewer #1.

*On page 11, authors claim that Dhh inactivation at the BBB drives endothelial and astrocytic activation. Since they did not identify astrocytic activation in the gray matter (figure 3, panels H and K), this implies that there is no BBB leakage in the gray matter but this is not shown. Otherwise, this conclusion shall be toned down.*

The conclusion of the results obtain in Fig 3 has been toned down. We concluded that, in the white matter, the BBB permeability induced by endothelial-specific Dhh inactivation drives astrocyte and endothelial reactivity.

The manuscript has been edited (Line 235 and Line 252) to clarify the results in Fig 3.

*In general the quality of immunofluorescence images is rather low and should be improved.*

We had to reduce the large file size to proceed to the submission of the manuscript which explains the quality loss of the immunofluorescence images. We do apologize for that. High quality images are available and can be sent to Reviewer #1.

*Reviewer #2, Julie Siegenthaler: This is a revised version of Hollier et al probing the molecular basis of a 2nd barrier system formed by the glial endfeet in response to neuroinflammation. The authors have done an excellent job addressing my previous comments, including greatly improved or completely new quantitative analysis (GFAP in SC sections Fig. 3, IgG leakage in perivascular spaces Fig. 4/Supp Fig. 4, Fig. 7), also adding a helpful diagram to bring the data in non-disease vs EAE together for the reader. The authors also added new discussion points requested that are important to understand 1) is BBB breakdown a universal inducer of Cldn4 and 'strengthening' the glial limitans, 2) is the induction of Cldn4 specific to VEGFA-mediated disruption or more broadly applicable to other cytokines and 3) is this a MS/EAE phenomenon.*

*I did note a typo in Figure 3J should be 'White' and the text in the summary figure is rather small, enlarging would help.*

The typo in Fig 3 Panel J has been corrected and the text from Fig 8 has been enlarged.

*I read through the other reviewer comments and the authors' response and I want to commend the authors for their efforts in addressing what were VERY extensive comments, especially considering lab shut downs and general pandemic related upheaval. Great job!*

We would like to thank Julie Siegenthaler for her very kind comments; we very much appreciate her support!

*Reviewer #3: The authors have thoroughly answered all queries. They should however add the source of the VE-cadherin-CreERT2 transgenic mouse in M&M.*

The source of the VE-cadherin-CreERT2 transgenic mouse has been specified in the Methods section of the manuscript. The sentence “The *Cdh5-Cre^ERT2^* mice and C57BL/6 mice were purchased from Jackson Laboratories (Bar Harbor, ME, USA).” has been added to the Methods section of the manuscript (Line 635 to Line 636).

Bibliography

1.	Okun E, Mattson MP, Arumugam TV. Involvement of Fc Receptors in Disorders of the Central Nervous System. Neuromolecular Med. juin 2010;12(2):164‑78.

2.	Nitta T, Yagita H, Sato K, Okumura K. Expression of Fc gamma receptors on astroglial cell lines and their role in the central nervous system. Neurosurgery. juill 1992;31(1):83‑7; discussion 87-88.
